# Viscoelastic Behavior of Cellular Biomaterials Based on Octet-Truss and Tetrahedron Topologies

**DOI:** 10.3390/ma17235865

**Published:** 2024-11-29

**Authors:** Reza Hedayati, Mohammad Shokrnia, Melikasadat Alavi, Mojtaba Sadighi, Mohammad Mohammadi Aghdam

**Affiliations:** 1 Aerospace Materials and Structures Department, Faculty of Aerospace Engineering, Delft University of Technology (TU Delft), Kluyverweg 1, 2629 HS Delft, The Netherlands; 2 Department of Mechanical Engineering, Amirkabir University of Technology (Tehran Polytechnic), Hafez Ave, Tehran 15916-34311, Iran; shokrnia@aut.ac.ir (M.S.); melika.alavi@aut.ac.ir (M.A.); mojtaba@aut.ac.ir (M.S.); aghdam@aut.ac.ir (M.M.A.)

**Keywords:** cellular biomaterials, viscoelastic properties, homogenization, porous material, asymptotic

## Abstract

Cellular biomaterials offer unique properties for diverse biomedical applications. However, their complex viscoelastic behavior requires careful consideration for design optimization. This study explores the effective viscoelastic response of two promising unit cell designs (tetrahedron-based and octet-truss) suitable for high porosity and strong mechanics. The asymptotic homogenization (AH) method was employed to determine effective longitudinal and shear moduli, as well as Poisson’s ratio, across various relative densities. Finite element simulations (ABAQUS) validated the AH results, demonstrating good agreement (<10% discrepancies). Additionally, analytical models and compression tests on 3D-printed lattice structures supported the theoretical predictions. The study revealed a strong correlation between relative density and the effective modulus of both designs. Notably, the tetrahedron-based design exhibited superior modulus, making it favorable for high loading levels, particularly when used as a high-density configuration. Both designs demonstrated minimal time-dependent elastic modulus changes and a near-constant Poisson’s ratio (0.34–0.349 for octet-truss, 0.316–0.326 for tetrahedron) across a 5–50% relative density range. While minimal, time-dependent modulus reduction needs to be considered in longer-term simulations ($t>10^{7}$ s). This study provides valuable insights into the viscoelastic behavior of these unit cells using the homogenization method, with potential applications in various biomedical fields.

## 1. Introduction

Cellular materials, characterized by their high surface-area-to-volume ratio, are ideal candidates for biological and medical applications. This unique characteristic fosters cell growth and enables tissue ingrowth within their internal spaces [1,2,3,4]. These biomaterials are specifically designed to interact with biological systems [5]. Their diverse applications in the biomedical field include biocompatible spinal implants, often fabricated from high-strength metals and polymers [6], which are commonly employed in bone replacement [7]. Additionally, cardiovascular devices such as heart valves can be manufactured using biomaterials to restore proper blood flow [8].

In tissue engineering, cellular structures serve as scaffolds to support tissue generation [9]. Given the critical role of these structures in various applications, particularly in medical devices, understanding their mechanical properties—especially the viscoelastic behavior inherent to polymeric implants—is essential. This knowledge is vital to ensure tissue compatibility, prevent structural deformation, and facilitate proper integration and functionality [10,11,12,13].

The optimal design of these cellular structures includes considerations such as porosity, pore size, elasticity, and viscoelastic modulus. For instance, bone scaffolds typically require porosity levels between 50–90% to maximize void volume for tissue growth [9]. Larger pores provide greater volume for nutrient transport and vascular growth, while smaller pores offer more surface area and promote faster tissue filling. Optimal pore sizes for bone are typically in the range of 200 µm to 1 mm [14].

However, despite the extensive research on the elastic properties of cellular structures [15,16,17,18,19], studies on their viscoelastic behavior remain limited. This gap is significant, as viscoelastic behavior is essential for understanding the time-dependent mechanical response of polymeric implants.

One of the first works by Biot [20] described the behavior of a viscoelastic porous material containing a viscous fluid, providing a general solution for non-homogeneous structures. Later works, such as those by Barbero and Luciano [21], presented analytical relationships in Laplace space for the relaxation modulus of composites with a viscoelastic matrix and transversely isotropic elastic fibers. Yi et al. [22] established a systematic method for determining the effective relaxation modulus of periodic viscoelastic composites through asymptotic homogenization (AH). They further employed topology optimization to improve stiffness and damping properties in these materials [23]. Liu et al. [24] and Tran et al. [25] employed homogenization methods to derive the linear viscoelastic properties of composites. In this study, elastic relationships were converted into viscoelastic relations using correspondence principle.

The recent research on the viscoelastic behavior of heterogonous materials has been largely confined to composite structures [26,27,28]. These studies have employed various methods, including homogenization and finite element analysis (FEA), to obtain their effective properties. Ramos et al. [27] utilized AH to calculate the properties of viscoelastic composites reinforced with fibers. Similarly, Glaesener et al. [28] analyzed truss-based beam structures using viscoelasticity theory. By assuming a continuum solid and applying a homogenization method, they derived the continuum form of the solution. These studies underscore the widespread use of homogenization methods for effective property analysis.

When it comes to applying AH to lattice structures, the foundational work by Bensoussan et al. [29] introduced multiscale methods for solving partial differential equations in a practical, accessible manner. Building on this, Guedes and Kikuchi [30] expanded the field by using AH to determine the effective elastic properties of composites while accounting for their microstructure. They also developed an optimized finite element method (FEM) for improved solution accuracy. Kalamkarov et al. [31] proposed analytical solutions for single-cell problems using AH and deriving formulas for the effective elastic moduli of various structures, including thin-walled composites, sandwich structures, and random composites. Similarly, Dinh et al. [32] proposed a numerical approach to predict the mechanical behavior of knitted fabrics, employing periodic boundary conditions to reduce computational costs and using homogenization to derive macroscopic behavior from unit cell studies. While Chen et al. [33] evaluated two homogenization techniques—fast Fourier transform (FFT) and asymptotic methods—for periodic composites, their study neglected the effects of time and viscoelasticity, leaving a gap in understanding time-dependent behaviors.

Despite significant advancements in understanding the elastic properties of lattice structures [34,35], there is still a notable absence of studies applying the AH method to the viscoelastic behavior of lattice structures. For example, in our recent work, we studied the bilinear elastic response of various honeycomb- and auxetic-based polymeric lattice structures under dynamic loads using microstructure-based FEM [36]. Similarly, another study proposed a plastic kinematic-based FEM with a focus on various impact and microstructure effects on the elastic behavior of closed-cell metal foams constructed from different unit cell types [37].

Although homogenization-based FEM has been applied to a variety of representative volume element (RVE) lattice designs, these studies have also primarily focused on determining effective elastic properties [38]. Analytical frameworks have been developed for irregular auxetic [39] and novel zigzag inclined ligament auxetic lattice designs [40], but their focus was similarly limited to elasticity.

More importantly, investigations into the time-dependent behavior of key unit cells, such as octet-trusses and tetrahedron-based designs, remain scarce. For instance, despite extensively explored studies on the elastic properties of octet-truss structures, including the influence of strut deformation, lattice angles, and beam tapering on stiffness, strength, and energy absorption [41,42,43,44,45], these efforts lack the research on their viscoelastic behavior, which is a critical gap because body tissues, even rigid ones (e.g., bones), exhibit time-dependent mechanical responses. Consequently, evaluating the viscoelastic behavior of porous biomedical devices, such as body implants, is essential for ensuring their performance and compatibility under different loading conditions.

This study focuses on the viscoelastic modeling of lattice structures formed from octet-truss and irregular tetrahedron-based unit cells, which are promising candidates for biological applications, particularly as bone replacement materials in dental and spinal implants. These structures are also highly relevant for a variety of engineering applications due to their high-strength and stiff topologies dominated by stretching behavior [10,46,47].

The octet-truss design is characterized by its high strength-to-weight ratio, excellent stiffness, and adaptability to varying loads. It has been extensively used in bone replacement materials, scaffolds for tissue engineering, orthopedic applications, and lightweight structural components [48]. This design demonstrates superior adaptability to bone elasticity, enhanced interconnectivity, and excellent support for bone growth. Conversely, tetrahedron-based unit cells exhibit remarkable compatibility and mechanical stability, with properties less influenced by changes in orientation or geometry [49]. These attributes make them highly reliable for applications requiring consistent performance under complex or multiaxial loading conditions. Clinical studies on implants as bone replacements in dogs, using porous implants made from octet-truss and tetrahedron-based unit cells, confirmed their excellent bone ingrowth within the femoral implant [1]. Additionally, design optimizations of these unit cells have shown their superiority over conventional cellular structures in terms of elasticity, specific energy absorption, and mechanical robustness [50,51]. Polyether ether ketone (PEEK), known for its biocompatibility, corrosion resistance, toughness, and durability, will serve as the base material for these structures.

In this study, we employ the AH method, particularly suited for lattice designs, to evaluate viscoelastic mechanical properties. This approach enables us to determine the impact of relative density on key properties, such as viscoelastic modulus and Poisson’s ratio, independent of the microstructure.

Five different relative densities were considered for each unit cell type. Time-dependent behavior was specifically explored, and the results were compared with finite element simulations conducted in ABAQUS. To further ensure the accuracy of our findings, 3D-printed lattice structures made from polylactic acid (PLA), chosen for its availability [52], were manufactured and subjected to compressive tests to validate the results of our work. Finally, a comparison between our results and findings from the previous research is conducted.

## 2. Materials and Methods

### 2.1. Designs

This research investigates the mechanical properties of two open-cell unit cell designs suitable for biomaterial applications: octet-truss and tetrahedron-based unit cells. Each unit cell type was constructed with five distinct relative density levels: 5%, 10%, 18.5%, 30%, and 50%.

**Figure 1:**
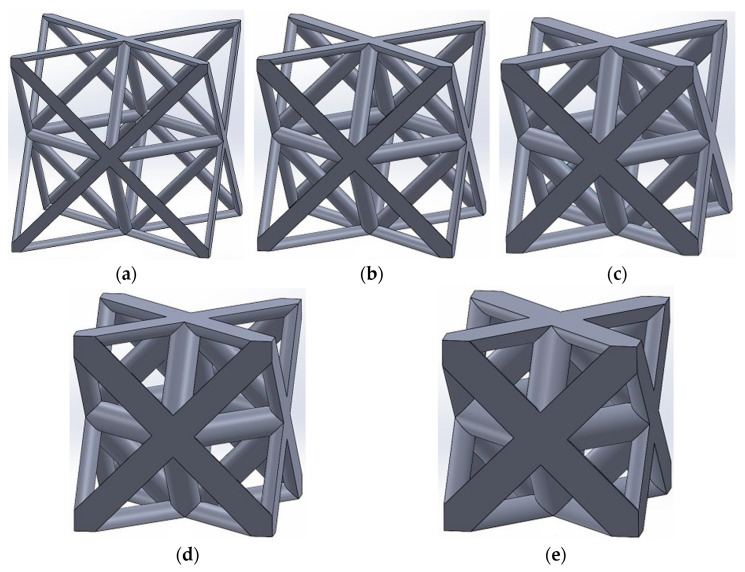
Octet-truss unit cells with relative densities of (**a**) 5% (r/a=0.031), (**b**) 10% (r/a=0.043), (**c**) 18.5% (r/a=0.058), (**d**) 30% (r/a=0.075), and (**e**) 50% (r/a=0.097). The parameters r and a represent the radius of struts and side length of the unit cell, respectively.

The octet-truss unit cell, illustrated in Figure 1, comprises a regular octahedron core surrounded by eight regular tetrahedra, each connected to a single face of the core. This configuration yields stretch-dominated behavior, leading to superior strength, stiffness, and a remarkable strength-to-weight ratio, making it suitable for high-strength porous biomaterials, such as orthopedic implants [53].

**Figure 2:**
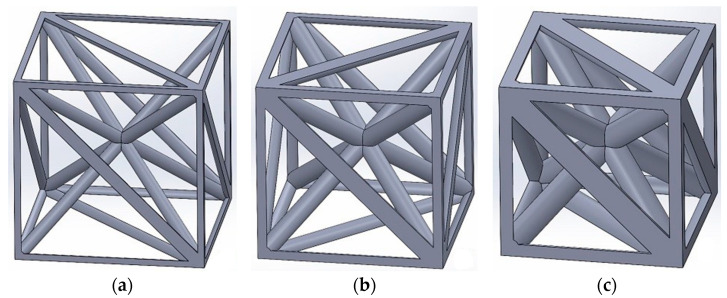

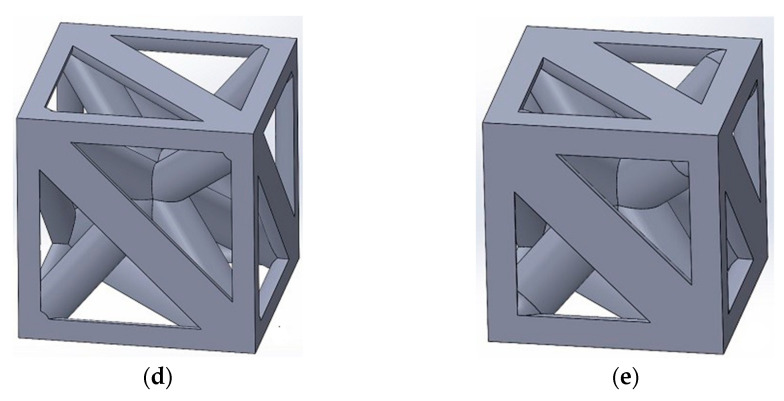
Tetrahedron-based unit cell for relative densities of (**a**) 5% (r/a=0.034), (**b**) 10% (r/a=0.050), (**c**) 18.5% (r/a=0.071), (**d**) 30% (r/a=0.094), and (**e**) 50% (r/a=0.128). The parameters r and a represent the radius of struts and side length of the unit cell, respectively.

On the other hand, tetrahedron-based unit cells utilize 12 Somerville’s irregular tetrahedron number 3 (details in Goldberg’s work [54]) arranged in a cubic configuration, as depicted in Figure 2. Table 1 summarizes the key properties of these tetrahedra. Notably, the listed angles represent the values at intersections of adjacent faces sharing a common side when forming a tetrahedron from the three vertices of B.

These tetrahedron-based unit cells are capable of conforming to intricate three-dimensional spaces with irregular borders and surfaces, required for applications in living organisms. The prior research [1,55,56] supports their suitability for biomaterial applications.

Both octet-truss and tetrahedron-based unit cells exhibit stretch-dominated behavior due to their internal connections and member arrangements [57,58]. This characteristic leads to superior structural efficiency compared to geometries where members undergo bending [59]. The strength of a stretch-dominated cell scales proportionally to ${\bar{\rho }}^{1.5}$, while that of a bending-dominated cell scales with $\bar{\rho }$, where $\bar{\rho }$ represents the relative density of the cellular structure. Since $\bar{\rho }$ is always less than one, the strength of a stretch-dominated geometry is inherently greater than that of a bending-dominated one for all porosity values. Similar reasoning applies to stiffness. Furthermore, due to the presence of symmetry planes and axes, the mechanical properties of these two geometries can be closely approximated by those of an isotropic material by selecting appropriate principal axes.

### 2.2. Material Model

**Figure 3:**
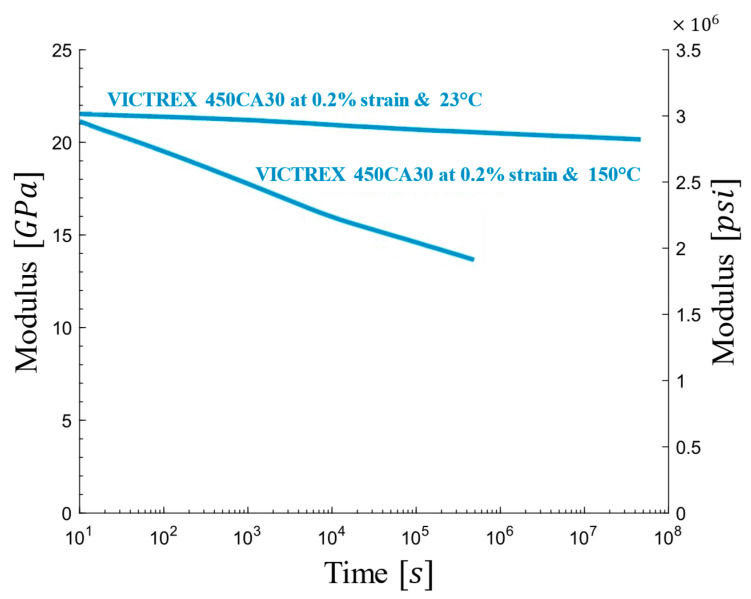
Experimental relaxation modulus curve of PEEK polymer with 30% carbon fiber at 23 °C and 150 °C [61].

PEEK polymer exhibits remarkable strength, toughness, and high-temperature resistance, making it a prime candidate for demanding biomaterial applications, particularly orthopedic implants. Reinforcing PEEK with carbon fiber reinforced polymer (CFRP) further enhances these properties, making the composite ideal for applications requiring superior longitudinal strength. Notably, CFRP with 30% content demonstrates exceptional performance, while also being biocompatible and readily processable. Figure 3 presents the experimental relaxation modulus curve of PEEK polymer with 30% carbon fiber, provided by Victrex, at 150 °C and room temperature [60]. The room temperature data are particularly relevant for biomaterial applications due to their closer resemblance to in vivo conditions.

The relaxation modulus, E(t), of the PEEK–carbon fiber composite was obtained using the Prony series model based on Figure 3, as shown in Equation (1):

(1) $$E\left(t\right)=19.8+1.85e^{-10^{-4}t}$$

Although developing a more detailed relationship with additional terms is possible, incorporating such models into homogenization-based FEM significantly increases computational time. Additionally, the lack of readily available direct shear modulus data for the PEEK–carbon fiber composite necessitated their estimation using established equations (Equations (2)–(4)) from prior studies. The shear modulus $G\left(t_{n}\right)$ rely on the calculated longitudinal relaxation modulus (E(t)) [61,62]:

(2) $$G\left(t_{n}\right)=\frac{\left[6K-G\left(t_{0}\right)]E\left(t_{n}\right)+G(t_{0})E(t_{n}-t_{1}\right)}{18K-3E\left(t_{0}\right)+E(t_{n}-t_{n-1})}+\frac{\sum_{i=1}^{n-1}G\left(t_{i}\right)[E\left(t_{n}-t_{i+1}\right)-E\left(t_{n}-t_{i-1}\right)]}{18K-3E\left(t_{0}\right)+E(t_{n}-t_{n-1})}$$

where

(3) $$G\left(t_{0}\right)=\frac{3KE(t_{0})}{9K-E(t_{0})}$$

(4) $$G\left(t_{1}\right)=\frac{\left[6K-G\left(t_{0}\right)]E\left(t_{1}\right)+G(t_{0})E(t_{0}\right)}{18K-3E\left(t_{0}\right)+E(t_{1})}$$

where K is the bulk modulus of constituent material, $t_{0}$ is the initial reference time point, and $t_{1}$ is the time at the second time step (i.e., the characteristic relaxation time). The calculated relaxation modulus values for both the longitudinal (E(t)) and shear (G(t)) components of the PEEK–carbon fiber composite at various time intervals are presented in Table 2.

The estimated function for the shear modulus is presented in Equation (5):

(5) $$G\left(t\right)=7.02+0.61e^{-10^{-4}t}$$

### 2.3. Asymptotic Homogenization (AH)

**Figure 4:**
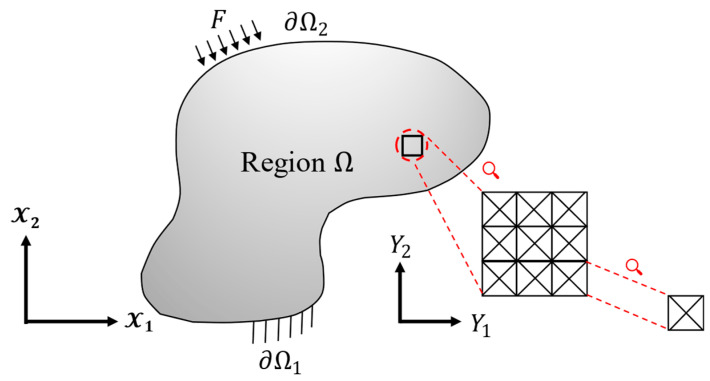
Schematic of the lattice structure, boundary conditions, and micro/macroscopic coordinate systems.

Asymptotic homogenization offers a framework for analyzing the mechanical response of composite materials by effectively bridging the gap between the intricate details of the microstructure (represented by the unit cell) and the overall macroscopic behavior of the material. This approach is based on the concept of two distinct spatial scales: Macroscopic Scale (x): This scale represents the larger one at which material behavior is typically observed; and microscopic Scale (y): This scale captures the finer details of the material’s microstructure. Figure 4 shows a schematic representation of these two scales.

The relationship between the macro and micro coordinates is established through a scaling parameter, η, in Equation (6) as [23]:

(6) $$y_{i}=\frac{x_{i}}{\eta }$$

The overall material behavior is then described by an asymptotic expansion of the displacement vector, $u_{i}^{\eta }\left(x,t\right)$, with respect to the scaling parameter η, as follows in Equation (7) [22,24]:

(7) $$u_{i}^{\eta }\left(x,t\right)=u_{i}^{0}\left(x,t\right)+\eta u_{i}^{1}\left(x,y,t\right)+\eta^{2}u_{i}^{2}\left(x,y,t\right)+\cdots$$

Here, $u_{i}^{0}\left(x,t\right)$ represents the macroscopic behavior, independent of the microscale details. The subsequent terms, $u_{i}^{n}$ (n>0), capture the local microscopic displacement variations. A key aspect of homogenization is the introduction of the homogenization tensor, $\chi_{i}^{kl}$. As shown in Equation (8), this tensor acts as a bridge between the macroscopic and microscopic displacements:

(8) $$u_{i}^{1}\left(x,y,s\right)=-\frac{\partial {\bar{u}}_{k}^{0}\left(x,s\right)}{\partial x_{l}}\chi_{i}^{kl}\left(y\right)+\varphi_{i}(x)$$

where $\frac{\partial {\bar{u}}_{k}^{0}\left(x,s\right)}{\partial x_{l}}$ denotes the spatial derivative of the macroscopic displacement with respect to the Laplace variable (s) in the frequency domain (common for viscoelastic analysis), and $\varphi_{i}(x)$ represents a particular solution arising from the homogenization process.

The homogenization tensor is determined by solving a system of partial differential equations derived from the requirement of minimizing the system’s potential energy. This minimization process leads to Equation (10), which relates the homogenized tensor to an arbitrary virtual displacement field, $v_{i}$.

#### 2.3.1. Viscoelastic Moduli

We now focus on determining the homogenized viscoelastic moduli using asymptotic homogenization. The detailed solution process involves solving a system of partial differential equations under periodic boundary conditions on the unit cell domain (y). This process allows us to obtain the components of the $\chi_{i}^{kl}$ tensor, which are crucial for calculating the overall stiffness (homogenized moduli) of the composite material in three dimensions.

The homogenized relaxation modulus is denoted by ${\bar{E}}_{ijkl}^{h}$. Considering the calculated homogenized viscoelastic modulus [22,23] and the fact that |Y| represents the entire unit cell volume (encompassing both the solid and void space), the Equations (9) and (10) are valid:

(9) $$s{\bar{E}}_{ijkl}^{h}\left(s\right)=\frac{1}{\left|Y\right|}\int_{Y}\left(s{\bar{E}}_{ijkl}\left(y,s\right)-s{\bar{E}}_{ijmn}\left(y,s\right)\frac{\partial \chi_{m}^{kl}\left(y\right)}{\partial y_{n}}\right)dy$$

(10) $$\begin{array}{l} \int_{Y}s{\bar{E}}_{ijmn}\left(y,s\right)\frac{\partial \chi_{m}^{kl}\left(y\right)}{\partial y_{n}}\frac{\partial v_{i}}{\partial y_{j}}dy=\int_{Y}s{\bar{E}}_{ijkl}\left(y,s\right)\frac{\partial v_{i}}{\partial y_{j}}dy;\\ i,j,k,l,m,n=1,2,3 \end{array}$$

Manipulating indices in Equation (10) yields distinct equations for $\chi_{m}^{kl}$ components, crucial for calculating the overall stiffness (homogenized modulus) of the material in three-dimensional space (represented by indices 1–3). Varying k and l defines distinct cases for $\chi_{m}^{kl}$ (index m is a summation index). Due to the symmetry of the $\chi^{kl}$ tensor, only 6 out of a total of 27 components in the full tensor are truly independent in a 3D isotropic material. This means that certain components of the tensor are interchangeable without affecting the overall behavior.

Equation (11) depicts it for *k* = *l* = 1:

(11) $$\int_{Y}s\;\langle \frac{\partial v_{1}}{\partial y_{1}}\frac{\partial v_{2}}{\partial y_{2}}\frac{\partial v_{3}}{\partial y_{3}}\left(\frac{\partial v_{1}}{\partial y_{2}}+\frac{\partial v_{2}}{\partial y_{1}}\right)\left(\frac{\partial v_{1}}{\partial y_{3}}+\frac{\partial v_{3}}{\partial y_{1}}\right)\left(\frac{\partial v_{2}}{\partial y_{3}}+\frac{\partial v_{3}}{\partial y_{2}}\right)\;\rangle \;\left[\bar{D}\right]{\langle \;\frac{\partial \chi_{1}^{11}}{\partial y_{1}}\frac{\partial \chi_{2}^{11}}{\partial y_{2}}\frac{\partial \chi_{3}^{11}}{\partial y_{3}}\left(\frac{\partial \chi_{1}^{11}}{\partial y_{2}}+\frac{\partial \chi_{2}^{11}}{\partial y_{1}}\right)\left(\frac{\partial \chi_{1}^{11}}{\partial y_{3}}+\frac{\partial \chi_{3}^{11}}{\partial y_{1}}\right)\left(\frac{\partial \chi_{2}^{11}}{\partial y_{3}}+\frac{\partial \chi_{3}^{11}}{\partial y_{2}}\right)\;\rangle }^{T}dy\;=\int_{Y}s\langle \;\frac{\partial v_{1}}{\partial y_{1}}\frac{\partial v_{2}}{\partial y_{2}}\frac{\partial v_{3}}{\partial y_{3}}\left(\frac{\partial v_{1}}{\partial y_{2}}+\frac{\partial v_{2}}{\partial y_{1}}\right)\left(\frac{\partial v_{1}}{\partial y_{3}}+\frac{\partial v_{3}}{\partial y_{1}}\right)\left(\frac{\partial v_{2}}{\partial y_{3}}+\frac{\partial v_{3}}{\partial y_{2}}\right)\;\rangle \;\left\{d_{1}\right\}dy$$

where [D] is a constant matrix containing material properties.

To solve problems within complex or arbitrary unit cell geometries, the solution domain needs discretization into smaller subdomains. This requires defining $\chi_{i}^{kl}$ (Equation (12)) and $v_{i}$ (Equation (13)) based on the shape function:

(12) $$\chi_{i}^{kl}=\sum_{m=1}^{n}N_{m}\chi_{i(m)}^{kl}$$

(13) $$v_{i}=\sum_{m=1}^{n}N_{m}v_{i(m)}$$

Shape function $\left(N_{m}\right)$ defines node number m. Moreover, n (number of nodes) and i (1–3) lead to 3 components each for $\chi_{i}^{kl}$ and $v_{i}$ per node. Each element has 12 degrees of freedom. The differential operator matrix is formed as in Equation (14):

(14) $$L=\left[\begin{array}{c} \begin{array}{ccc} \frac{\partial }{\partial y_{1}} & 0 & 0\\ 0 & \frac{\partial }{\partial y_{2}} & 0\\ 0 & 0 & \frac{\partial }{\partial y_{3}} \end{array}\\ \begin{array}{ccc} \frac{\partial }{\partial y_{2}} & \frac{\partial }{\partial y_{1}} & 0\\ \frac{\partial }{\partial y_{3}} & 0 & \frac{\partial }{\partial y_{1}}\\ 0 & \frac{\partial }{\partial y_{3}} & \frac{\partial }{\partial y_{2}} \end{array} \end{array}\right]$$

Therefore, Equation (11) can be written as Equation (15),

(15) $$\int_{Y}s\left[L][N_{m}\right]\left\{v_{m}\right\}\left[\bar{D}\right]\left[L][N_{m}\right]\left\{\chi_{m}\right\}dy=\int_{Y}s\left[L][N_{m}\right]\left\{v_{m}\right\}\left\{d_{1}\right\}dy$$

where $d_{1}$ represents the source term.

Multiplying $\left[L\right]$ and [$N_{m}$] yields the derivative matrix of $N_{m}$, denoted by *B* Equation (16). This matrix can then be substituted into Equation (15), resulting in Equation (17).

(16) $$B=L\cdot N_{m}$$

(17) $${\left[B\right]}^{T}\left[\bar{D}\right]\left[B\right]\left\{\chi_{m}\right\}={\left[B\right]}^{T}\left\{d_{1}\right\}$$

The equation relating the force and displacement using the stiffness matrix in the FEM is written as Equation (18):

(18) $$\left[K\right]\left\{X\right\}=\{F\}$$

By comparing Equations (17) and (18), the stiffness and force matrices for the problem are as follows, as shown in Equations (19) and (20):

(19) $${\left[k\right]}^{e}={{\left[B\right]}^{T}}^{e}\left[\bar{D}\right]{\left[B\right]}^{e}$$

(20) $${\left\{f\right\}}^{e}={{\left[B\right]}^{T}}^{e}\left\{d_{1}\right\}$$

Here, ${\left[k\right]}^{e}$ and $f^{e}$ represent the element stiffness matrix and element force vector, respectively.

To demonstrate that a force vector can represent an initial strain loading, the resulting nodal force is defined in Equation (21) [63].

(21) $$\{f_{i}^{\varepsilon^{0}}\}=\int_{Y(e)}{{\left[B\right]}^{T}}^{e}D\varepsilon^{0}dy$$

This force induces the initial strain (Equation (22)):

(22) $$D\varepsilon^{0}=d_{1}$$

where $\varepsilon^{0}$ represents the initial strain vector (See Equation (23)).

(23) $$\varepsilon_{11}^{0}=1;\;\varepsilon_{22}^{0}=0;\;\varepsilon_{33}^{0}=0;\;\varepsilon_{12}^{0}=0;\;\varepsilon_{13}^{0}=0;\;\varepsilon_{23}^{0}=0$$

After assembling the stiffness and force matrices, the three unknown components of the problem, $\chi_{i}^{11}$, are calculated. This allows for the determination of the three components of the homogenized relaxation modulus. Finally, the inverse Laplace transform (Equation (10)) is applied to each calculated component, as derived in Equations (24)–(26):

(24) $${\bar{E}}_{1111}^{h}\left(s\right)=\frac{1}{\left|Y\right|}\int_{Y}\left[{\bar{E}}_{1111}\left(y,s\right)-\left({\bar{E}}_{1111}\left(y,s\right)\frac{\partial \chi_{1}^{11}\left(y\right)}{\partial y_{1}}+{\bar{E}}_{1122}\left(y,s\right)\frac{\partial \chi_{2}^{11}\left(y\right)}{\partial y_{2}}+{\bar{E}}_{1133}\left(y,s\right)\frac{\partial \chi_{3}^{11}\left(y\right)}{\partial y_{3}}\right)\right]dy$$

(25) $${\bar{E}}_{2211}^{h}\left(s\right)=\frac{1}{\left|Y\right|}\int_{Y}\left[{\bar{E}}_{2211}\left(y,s\right)-\left({\bar{E}}_{2211}\left(y,s\right)\frac{\partial \chi_{1}^{11}\left(y\right)}{\partial y_{1}}+{\bar{E}}_{2222}\left(y,s\right)\frac{\partial \chi_{2}^{11}\left(y\right)}{\partial y_{2}}+{\bar{E}}_{2233}\left(y,s\right)\frac{\partial \chi_{3}^{11}\left(y\right)}{\partial y_{3}}\right)\right]dy$$

(26) $${\bar{E}}_{3311}^{h}\left(s\right)=\frac{1}{\left|Y\right|}\int_{Y}\left[{\bar{E}}_{3311}\left(y,s\right)-\left({\bar{E}}_{3311}\left(y,s\right)\frac{\partial \chi_{1}^{11}\left(y\right)}{\partial y_{1}}+{\bar{E}}_{3322}\left(y,s\right)\frac{\partial \chi_{2}^{11}\left(y\right)}{\partial y_{2}}+{\bar{E}}_{3333}\left(y,s\right)\frac{\partial \chi_{3}^{11}\left(y\right)}{\partial y_{3}}\right)\right]dy$$

Following a similar approach for k, l combinations of 2, 2; 3, 3; 1, 2; and 1, 3 (as described in Table 3), all the remaining viscoelastic modulus components can be derived.

#### 2.3.2. Poisson’s Ratio

**Figure 5:**
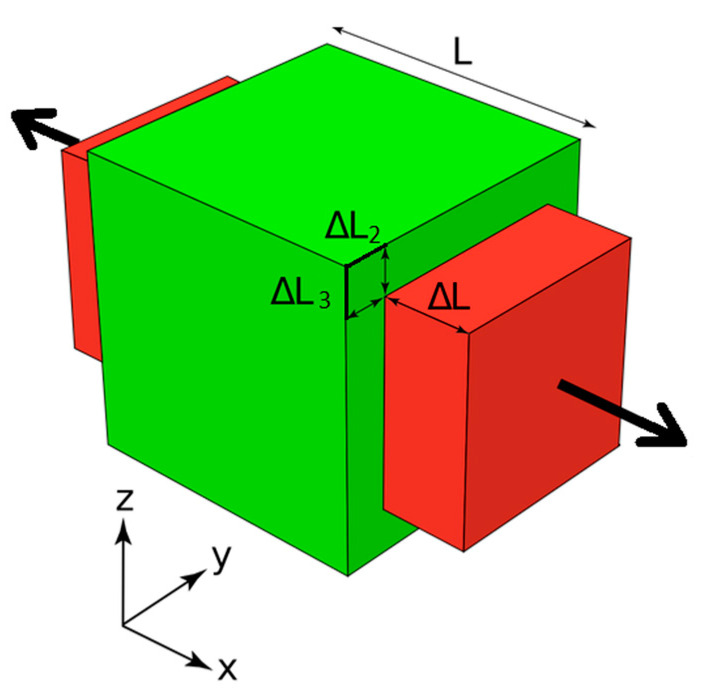
Deformation of material by longitudinal stress.

Leveraging the cubic geometry depicted in Figure 5, Poisson’s ratio can be determined using the following relationships in Equation (27):

(27) $$v_{12}=-\frac{\varepsilon_{22}}{\varepsilon_{11}}\;where\;\varepsilon_{11}=\frac{2∆L}{L},\varepsilon_{22}=\frac{{-2∆L}_{2}}{L}$$

Here, $\nu_{12}$ represents Poisson’s ratio in the 1–2 plane, $\varepsilon_{11}$ and $\varepsilon_{22}$ are the corresponding normal strains, ΔL is the change in length, and L is the original length. All other components of $\nu_{ij}$ can be obtained similarly, and for isotropic materials, these components are equal (See Equation (27)).

The lateral homogenization method is employed to determine the components of $\chi_{i}^{kl}$, which account for displacements under different boundary conditions. For instance, $v_{12}$ can be calculated for the first case problem (k=l=1).

### 2.4. Periodic Boundary Condition

Given the periodic nature of the lattice structure, characterized by the repeating unit cell, periodic boundary conditions are employed to efficiently derive equations for both unit cell types. These conditions relate parallel and opposite external boundary surfaces. The underlying assumption is that the displacement on one face of the unit cell replicates the displacement on the opposite face.

**Figure 6:**
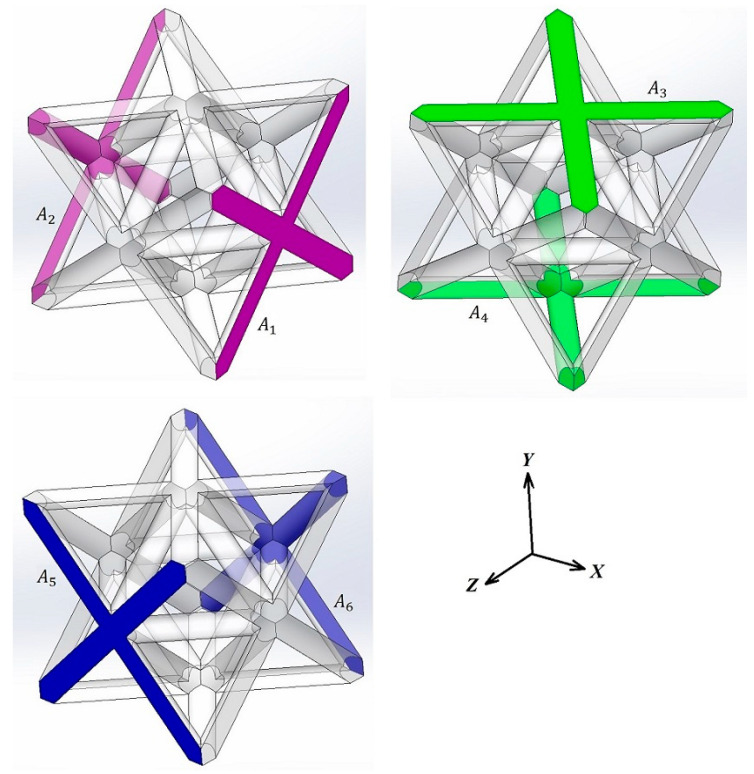
Opposite boundary surfaces in the octet-truss unit cell.

Figure 6 depicts the opposing surfaces of the octet-truss unit cell essential for applying periodic boundary conditions: A1–A2 (normal vector: x-direction), A3–A4 (normal vector: y-direction), and A5–A6 (normal vector: z-direction). Surfaces A1–A2, A3–A4, and A5–A6 define the unit cell boundaries where periodic conditions are applied. By enforcing the same displacement conditions on these opposing faces, we effectively eliminate the need to model the entire lattice structure and focus solely on the behavior within the unit cell.

The specific boundary conditions for $A_{1}$ and $A_{2}$ are provided in Equations (28)–(30):

(28) $$u_{1}^{A_{1}}=u_{1}^{A_{2}}+\varepsilon_{11}∆x_{1}$$

(29) $$u_{2}^{A_{1}}=u_{2}^{A_{2}}+\varepsilon_{21}∆x_{1}$$

(30) $$u_{3}^{A_{1}}=u_{3}^{A_{2}}+\varepsilon_{31}∆x_{1}$$

where $u_{i}$ represents the displacement component in the i-direction (i = 1, 2, 3), Δx is a reference length, and $\varepsilon_{ij}$ are the strain components. Similar conditions are established for the other two pairs of surfaces.

Several methods exist to incorporate boundary conditions into finite element solutions. Due to the constrained nature of this problem, the Lagrange multiplier method is best suited for enforcing these constraints [64].

### 2.5. Finite Element Modeling Using ABAQUS

To validate the results obtained from the asymptotic homogenization method, the finite element software ABAQUS (version 2022, Dassault Systèmes, Vélizy-Villacoublay, France) was employed. The boundary conditions used in the simulations were adopted from the study by Omairey et al. [65]. It is important to note that initially flat boundary surfaces may experience deformations and become curved upon loading [66].

A key challenge in this study was implementing the problem’s boundary conditions in ABAQUS. This process involved several steps, beginning with the identification of boundary nodes where the conditions would be applied individually. It is noteworthy that for both the AH and FE methods, tetrahedral elements were used to discretize the unit cells. These elements define the shape functions (explained in Section 2.3.1) used to solve for the stiffness and force matrices of each element. A more comprehensive analysis of the meshing approach is provided in the Online Supplementary Material accompanying the paper.

One of the most fundamental methods for analyzing the viscoelastic behavior of materials is the relaxation test, in which the material is subjected to a constant strain and held for a relatively long period. During this process, the stress values in different points of the material gradually decrease, a phenomenon known as stress relaxation. The relaxation test was simulated using ABAQUS by applying a 1% uniaxial constant strain with an initial time step of 1 s, followed by a time step of $10^{7}\;s$, to observe the material’s response over time. von Mises stress analysis has proven to be a valuable tool for understanding the behavior of viscoelastic materials, particularly their stress relaxation characteristics. In this method, the stress values recorded at the end of the first time step, immediately after the strain was applied, represent the elastic response of the material, while the stress values observed after a prolonged period correspond to the relaxation response.

### 2.6. Experiments

**Figure 7:**
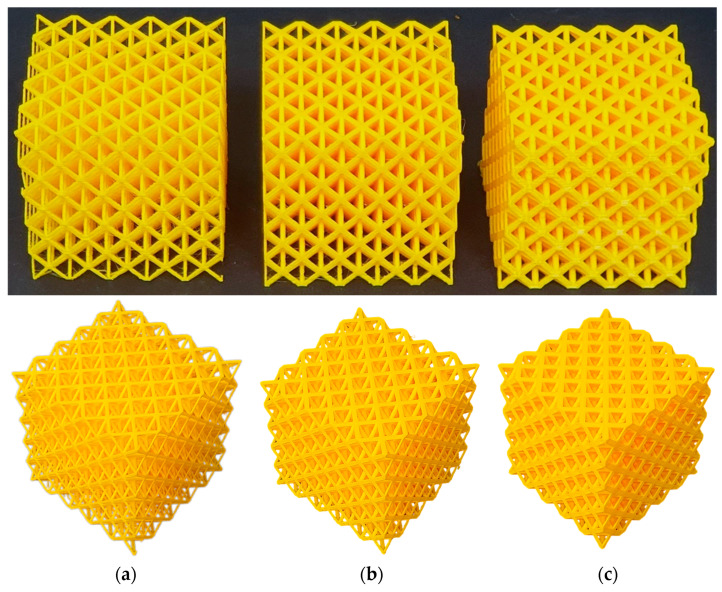
Octet-truss 3D printed lattice structures with relative densities of (**a**) 18%, (**b**) 30%, and (**c**) 50%.

For experimental validation of the numerical methods, octet-truss unit cell lattice structures were designed using SolidWorks (version 2022, Dassault Systèmes, Vélizy-Villacoublay, France) and fabricated using the fused deposition modeling (FDM) technique with PLA filament. To isolate the effect of relative density on the mechanical response, three octet-truss designs with relative densities of 18%, 30%, and 50% were manufactured using Creality Ender 3 3D printer (Shenzhen, China), see Figure 7. Each sample had consistent dimensions of 8 × 8 × 8 cm^3^. The nozzle diameter was 0.4 mm, and the printing temperature was set to 220 °C. A layer height and printing resolution of 0.2 mm was used. The Ultimaker Cura program was used to create gcodes necessary for the 3D printer.

**Figure 8:**
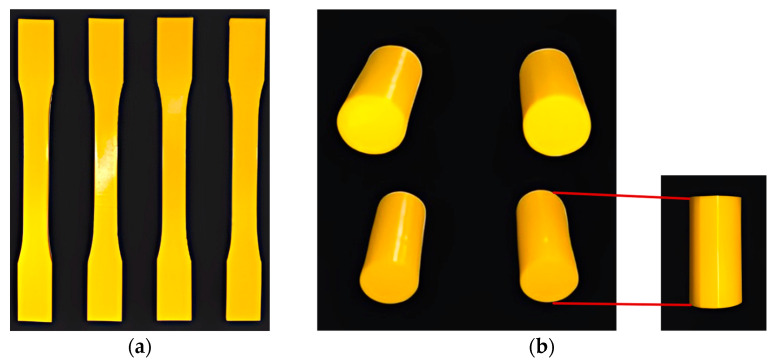
3D-printed (**a**) dog-bone and (**b**) cylindrical specimens made up of PLA for tensile and compression tests, respectively.

Prior to testing the lattice structures, the mechanical properties of the bulk PLA material were determined through tensile and compression tests on dog-bone and cylindrical specimens, respectively (See Figure 8). The cylindrical compression test specimens had a diameter of 12.7 mm and a height of 25.4 mm. The dimensions of the dog-bone specimen can be found in the Online Supplementary Materials accompanying the paper.

The fabricated lattice structures were then subjected to quasi-static compression tests at a strain rate of 1 s^−1^. This experimental validation aimed to confirm the theoretical predictions, particularly regarding the near-independence of the normalized homogenization modulus at t=0 from the bulk material properties.

## 3. Results and Discussion

This section comprehensively discusses the results obtained from the asymptotic homogenization method and the finite element simulations performed using ABAQUS software. The discussion focuses on both chosen unit cell geometries: the octet-truss and the tetrahedron.

### 3.1. Octet-Truss Unit Cell

**Figure 9:**
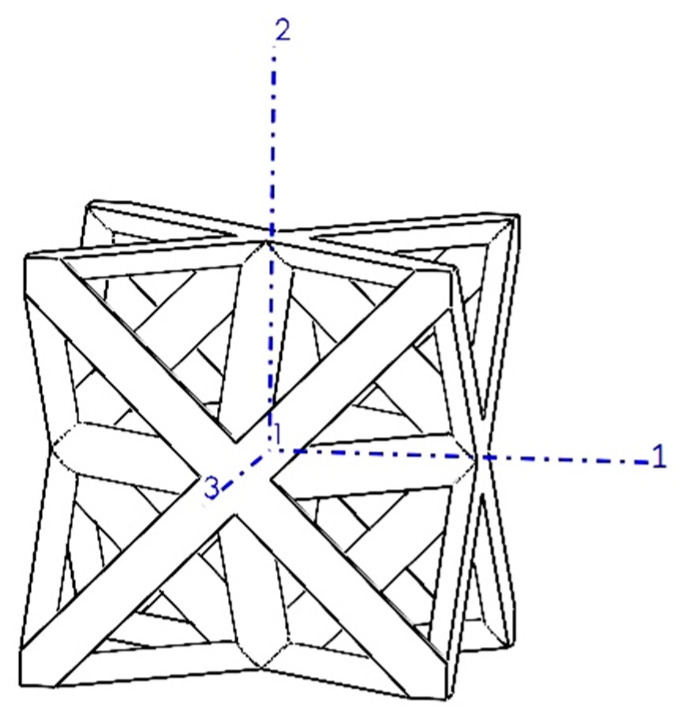
Octet-truss unit cell with a coordinate system aligned with its symmetry planes.

Due to the three axes of symmetry in the octet-truss unit cell (Figure 9), a coordinate system aligned with these axes results in a uniform geometry in all three main orthogonal directions (i.e., yielding an isotropic material). Consequently, the relaxation tensor, characterizing the material’s time-dependent stress response to strain, was simplified in this case. In an isotropic material case, the relaxation tensor reduced to only have two independent components: the longitudinal relaxation modulus E(t) and the shear relaxation modulus G(t) obtained from the material model (Section 2.2). This simplification was advantageous in terms of computational efficiency and interpreting the results.

#### 3.1.1. Quasi-Static Compressive Tests Results

**Figure 10:**
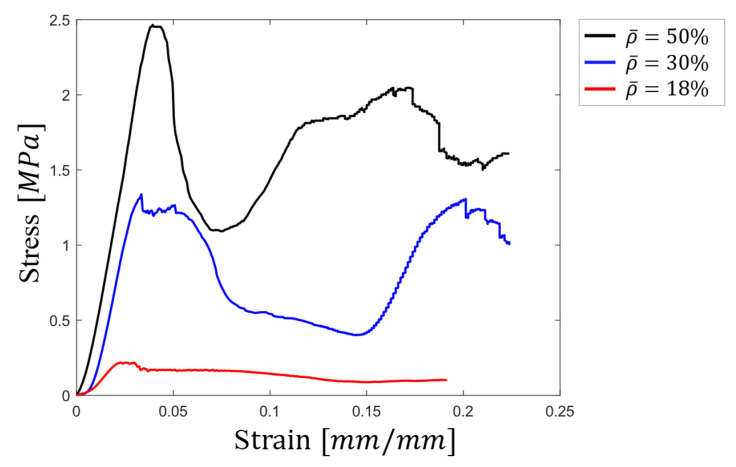
Stress–strain curve of octet-truss lattices with 18%, 30%, and 50% relative densities.

Figure 10 presents stress–strain curves obtained from quasi-static compression tests on octet-truss lattice samples made from PLA with three different relative densities (18%, 30%, and 50%). These curves highlight the significant impact of density on the mechanical properties of the structures.

The sample with the highest relative density (50%) exhibited a yield strength of approximately 2.47 MPa. This value was significantly higher than the 1.29 MPa and 0.2 MPa yield strengths observed for the samples with relative densities of 30% and 18%, respectively. This translated to a decrease in strength of roughly 48% and 90% for the 30% and 18% relative density samples compared to the 50% relative density sample, despite a density reduction of only 40% and 64%, respectively.

Elastic modulus, estimated from the slope of the linear region in the stress–strain curves, followed a similar trend. The values for the 50%, 30%, and 18% relative density samples were 74.9 MPa, 65.1 MPa, and 15.2 MPa, respectively. Interestingly, the decrease in elastic modulus from 50% to 30% density (which was 13%) was considerably smaller than the decrease in strength observed for the same comparison.

A noteworthy observation was the behavior of the stress–strain curves at higher strains for the samples with higher relative densities (50% and 30%). After a certain point (around 0.08 strain for the 50% relative density sample and 0.15 strain for the 30% relative density sample), the stress values began to increase again. This phenomenon, known as densification of porous materials, was not observed in the stress–strain curve of the lowest density sample (18%). Although the densification regime for the lowest-density structure was not observed in this graph, it is expected to be seen if the applied strain was beyond 0.2.

#### 3.1.2. Numerical Results

##### Viscoelastic Moduli

Table 4 presents the components ($E_{11}$ and $G_{12}$) of the relaxation tensor for the octet-truss unit cell, obtained using three methods: AH, FE, and the work of Deshpande et al. [67]. Results are presented for initial and long-term conditions across various relative densities. It is worth noting that 5% relative density is equivalent to 95% porosity.

Deshpande et al. [67] investigated the elastic behavior of the octet-truss, and their proposed relationship between elastic modulus components and relative density was limited to time-independent scenarios and low densities. In their derivations, Deshpande et al. neglect the bending deformation effects, resulting in less accurate results at high densities [67].

The relationship presented in the work of Deshpande et al. is as follows:

(31) $$C_{ijkl}=\bar{\rho }E_{s}\left[\begin{array}{cccccc} \frac{1}{6} & \frac{1}{12} & \frac{1}{12} & 0 & 0 & 0\\ \frac{1}{12} & \frac{1}{6} & \frac{1}{12} & 0 & 0 & 0\\ \frac{1}{12} & \frac{1}{12} & \frac{1}{6} & 0 & 0 & 0\\ 0 & 0 & 0 & \frac{1}{12} & 0 & 0\\ 0 & 0 & 0 & 0 & \frac{1}{12} & 0\\ 0 & 0 & 0 & 0 & 0 & \frac{1}{12} \end{array}\right]$$

where $\bar{\rho }$ is the relative density, and $E_{s}$ is the elastic modulus of the bulk material.

Based on the viscoelastic model defined previously, the AH method could yield time-dependent results. As shown in Equations (32) and (33), these results include approximations for the octet-truss’s longitudinal and shear modulus with respect to its density:

(32) $$E_{1111}\left(t\right)=0.507907+0.211945e^{\left(-1.016\times 10^{-4}t\right)}-0.126831e^{-10^{-4}t}$$

(33) $$G_{12}\left(t\right)=0.362232+0.040083e^{-10^{-4}t}$$

**Figure 11:**
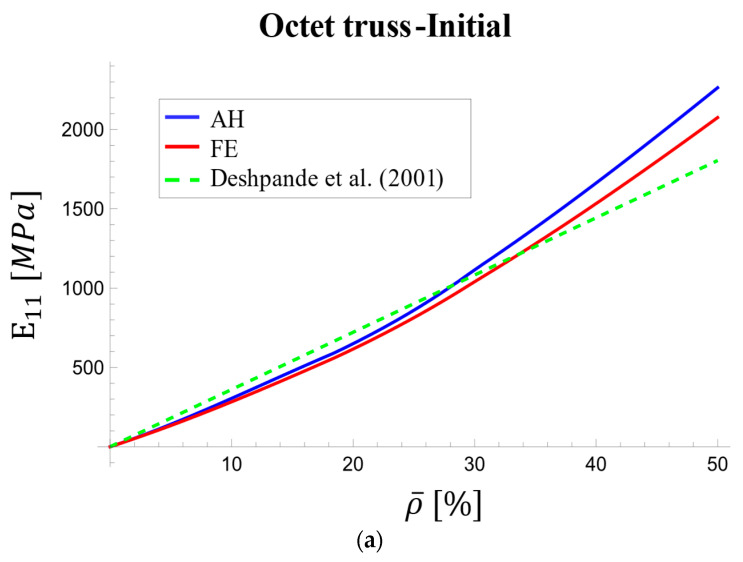

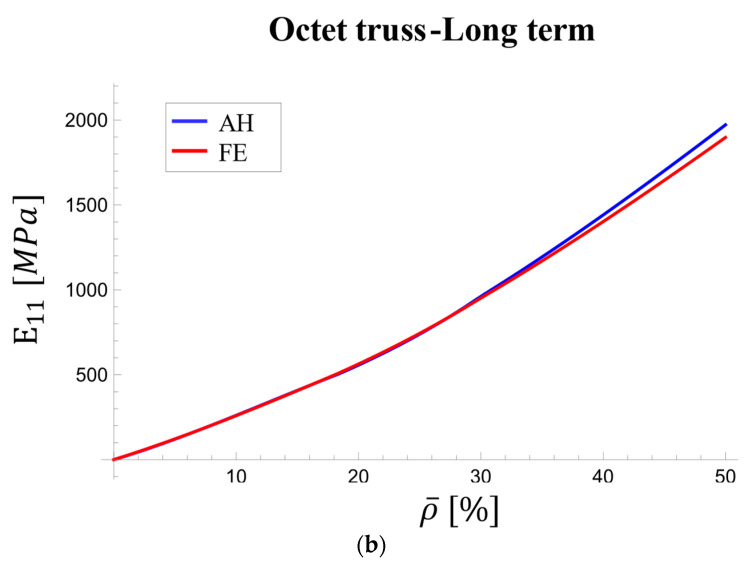
Variations in effective viscoelastic modulus with relative density [67]: (**a**) at initial stage and (**b**) in the long term.

Figure 11 presents the variations in the elastic modulus of an octet-truss unit cell with respect to its relative density at both the initial loading and long-term (t→∞) stages. These results were obtained from AH, FE, and the analytical model of Deshpande et al. (which is only applicable to the initial loading stage due to its focus on elastic behavior).

Figure 11b illustrates the nonlinear relationship between changes in the effective elastic modulus ($E_{1111}$) obtained from homogenization and FE analysis. The slope of these curves increased as relative density increased. Deshpande’s model, however, predicted a nearly linear trend. Notably, around 30% relative density, the Deshpande curve intersected the AH and FE curves, and the discrepancy widened at higher densities.

The difference between AH and FE results varied from 5.8% at 5% relative density to 8.3% at 50% relative density at the loading onset (Figure 11a). The long-term result (Figure 11b) showed better agreement at 50% density, with a maximum difference of 3.8%.

**Figure 12:**
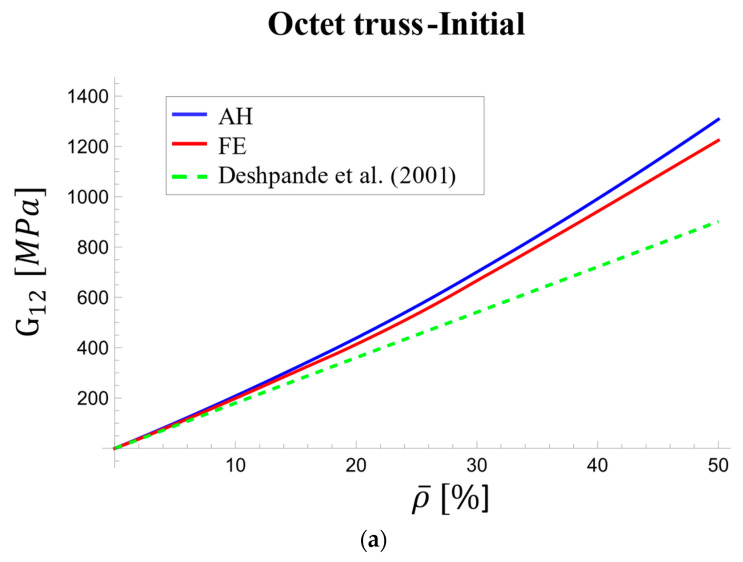

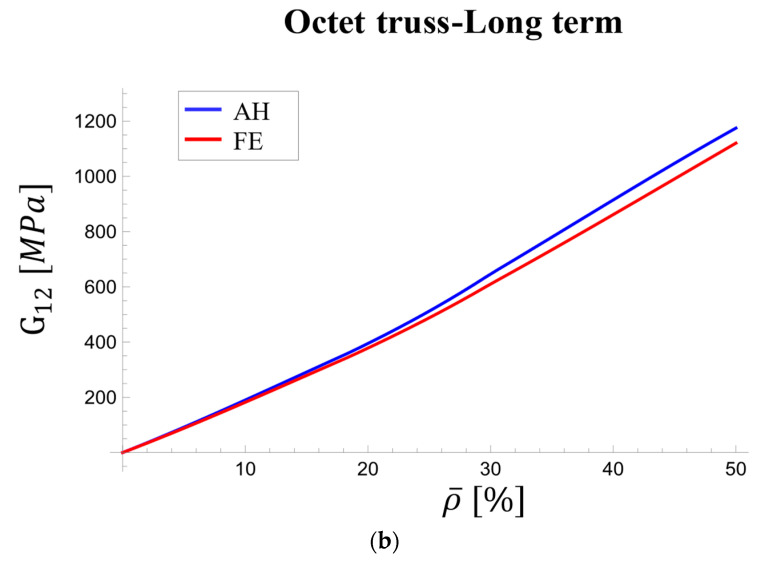
Variations of effective viscoelastic shear modulus with relative density [67]: (**a**) at initial stage and (**b**) in long term.

Similar to the elastic modulus, Figure 12 depicts the variations in the homogenized shear modulus of the octet-truss unit cell with respect to relative density at both initial loading and long-term stages. The discrepancy between homogenization and FE results, compared to Deshpande’s model, became more pronounced as the density increased.

Figure 12a shows a growing discrepancy between the Deshpande model and homogenization results at loading onset, increasing from 11% at 5% density to 31% at 50% density. Conversely, homogenization and FE analysis demonstrated strong agreement, with a maximum difference of only 6.3% at 50% density. In the long term (Figure 12b), the maximum difference between the two methods remained around 3%, indicating good agreement.

**Figure 13:**
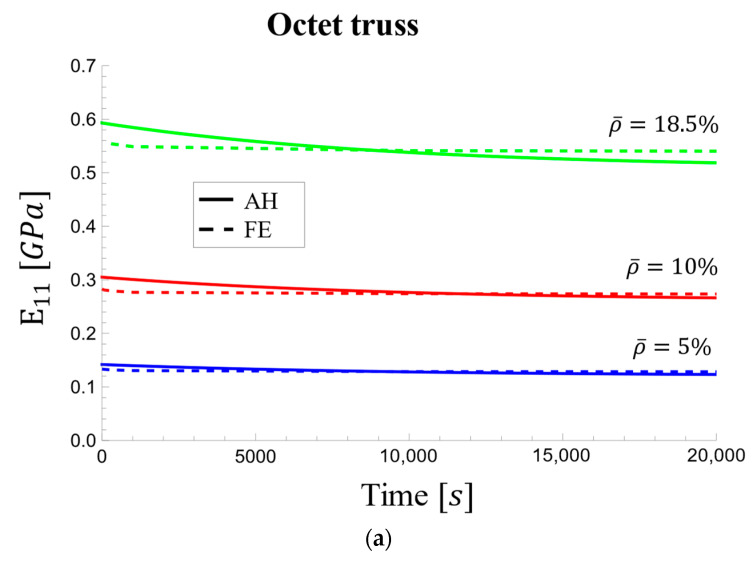

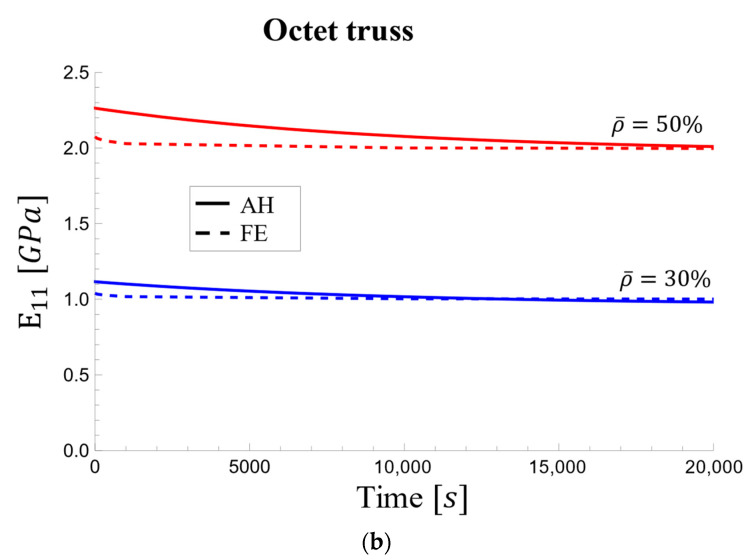
Viscoelastic effective elastic modulus changes over time: (**a**) for relative densities of 5%, 10%, and 18.5% and (**b**) for relative densities of 30% and 50%.

Figure 13 presents the variation in viscoelastic effective modulus over time. As expected, both methods showed a decrease in modulus, but the FE results exhibited a slower rate of decrease compared to the AH results. At lower densities, where the assumptions are more valid, the AH and FE curves for the elastic modulus intersected around 10,000 s. Interestingly, the AH method exhibited a faster convergence towards a constant value, while the FE curves continued to decrease at a slower rate even at longer durations due to its ability to capture more complex material behavior (FE) in the long run compared to the averaging nature of the AH. This initial difference at 50% density was around 3.8%, highlighting the modeling’s approach influence.

**Figure 14:**
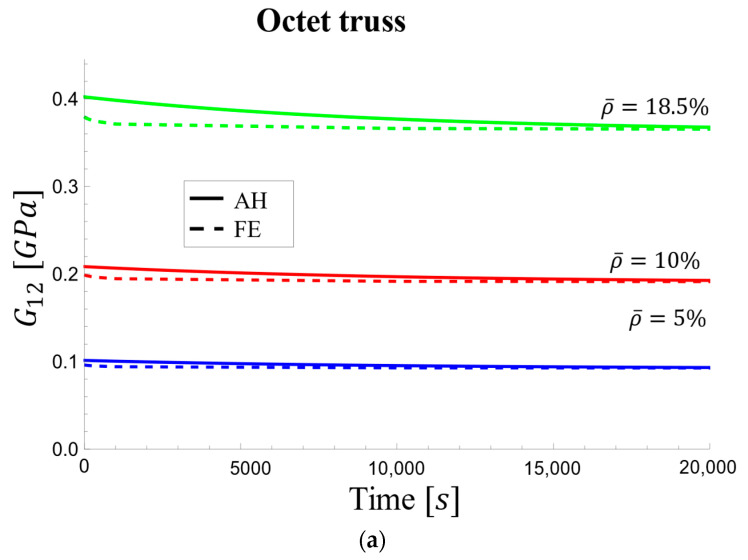

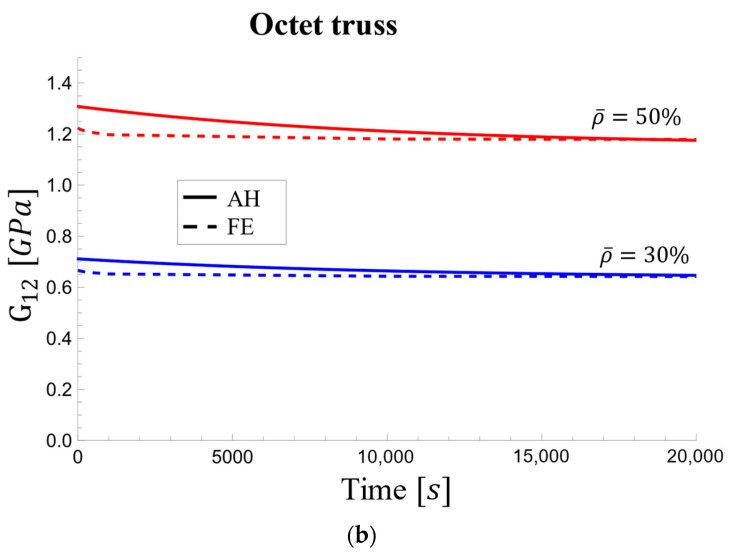
Viscoelastic effective shear modulus changes over time: (**a**) for relative densities of 5%, 10%, and 18.5% and (**b**) for relative densities of 30% and 50%.

The effective viscoelastic shear modulus values in Figure 14 decreased more sharply, potentially due to an approximation in the model. Despite this steeper decline, the AH and FE curves converged to similar values at approximately 5 h (20,000 s) across all densities. The initial difference between the curves was more pronounced at 50% density, reaching a maximum of 3.6%.

##### Poisson’s Ratio

**Figure 15:**
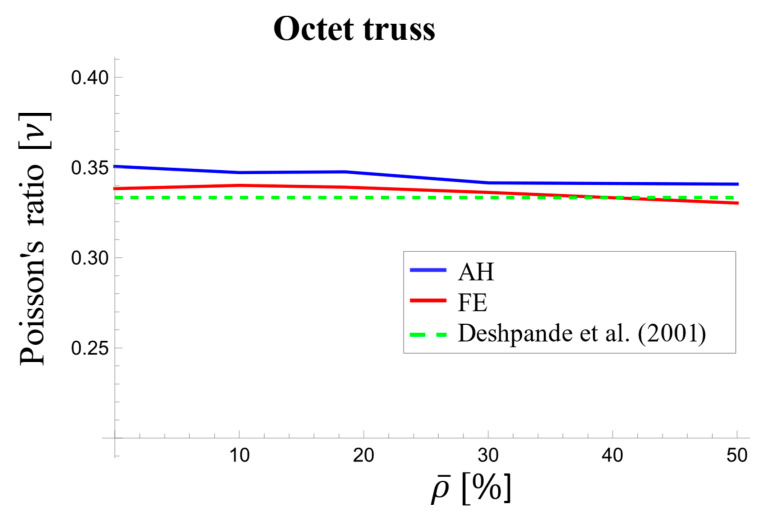
Variations in the effective Poisson’s ratio with respect to relative density at the beginning of loading for the octet-truss unit cell [67].

Table 5 compares the effective Poisson’s ratio for the octet-truss unit cell obtained from homogenization and FEM at both the initial loading stage and the long-term stage. Similar to the elastic and shear moduli, the octet-truss unit cell’s Poisson’s ratio displayed a dependence on relative density at the beginning of loading, although the dependence was relatively small (Figure 15). Unlike the AH and FE models, which showed a decrease in Poisson’s ratio value with increasing density, Deshpande’s model [67] predicted a constant value of 1/3, resulting in deviations of up to 4.5% from the AH method’s values. Both the AH and FE models exhibited consistent trends with density, as shown in Table 5 and Figure 14. The difference between these two models was from 1.5% to 3% for Poisson’s ratio at loading onset and remained within 1.6% to 3.3% in the long term.

**Figure 16:**
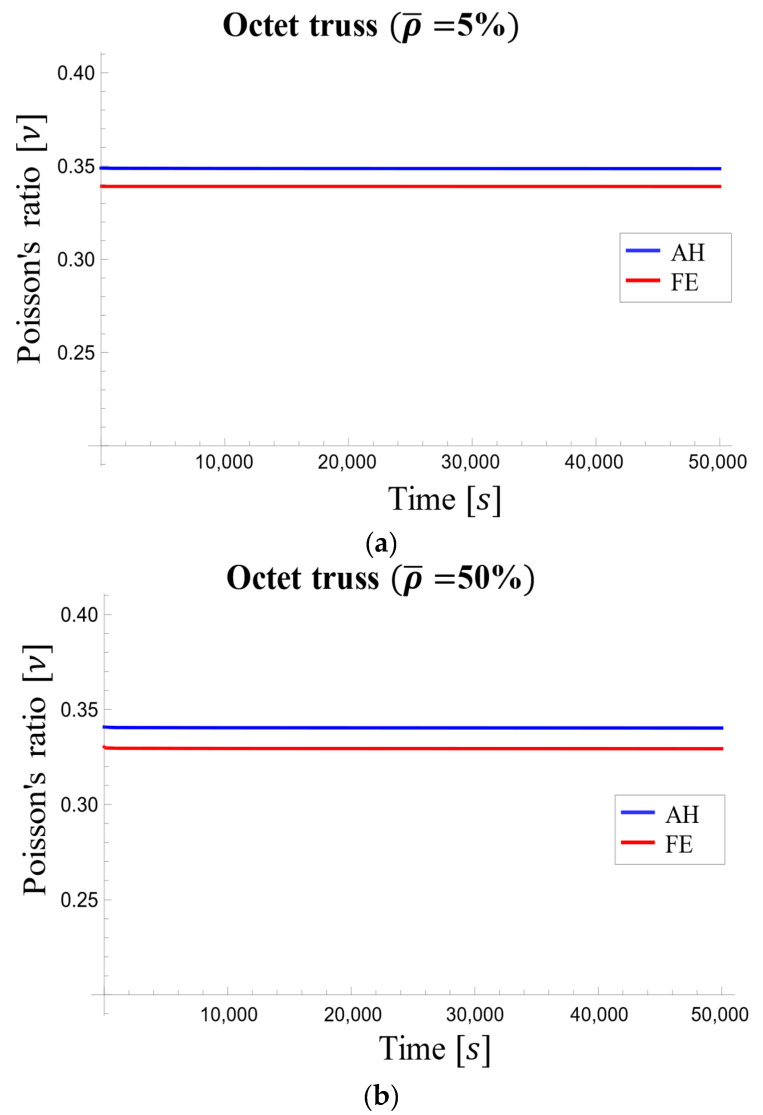
Poisson’s ratio changes in the octet-truss unit cell over time for relative densities of (**a**) 5% and (**b**) 50%.

Due to the slight sensitivity of Poisson’s ratio to time and relative density, we have shown the dependence of Poisson’s ratio to time only for structures with 5% and 50% densities in Figure 16. As shown, Poisson’s ratio displayed minimal variation across time for both presented densities.

### 3.2. Tetrahedron-Based

**Figure 17:**
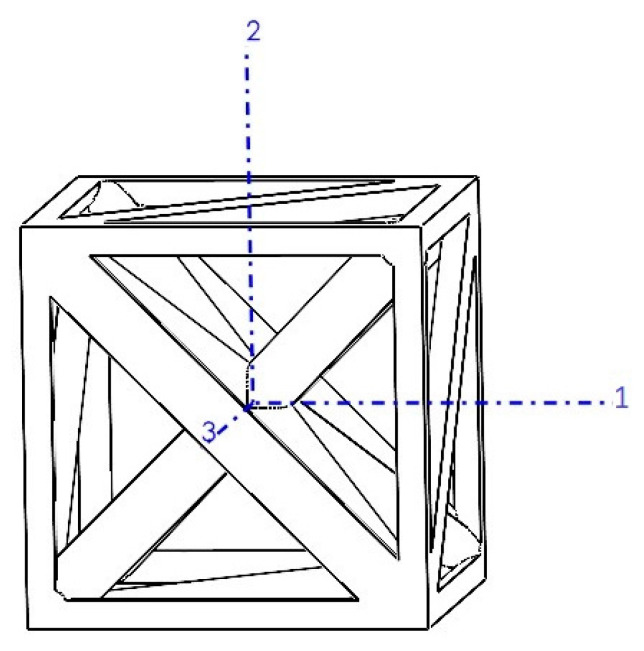
Tetrahedral unit cell with isotropic coordinate system.

Similar to the octet-truss, tetrahedral unit cells exhibit anisotropy. However, isotropy can be attained by strategically selecting a proper coordinate system (Figure 17). This results in uniform properties in all three orthogonal directions, allowing for the use of a single effective value for the longitudinal modulus, shear modulus, and Poisson’s ratio along all three axes (1, 2, and 3).

Table 6 lists viscoelastic tensor components ($E_{11}$ and $G_{12}$) for the tetrahedral unit cell obtained from both the AH and FE methods at initial time and after a long period.

The homogenization method yielded time-dependent results. The resulting relationship for the tetrahedral unit cell with a density of 18.5% is as follows:

(34) $$E_{1111}\left(t\right)=0.691326+0.254104e^{-1.016\times 10^{-4}\;t}-0.141560e^{-10^{-4}\;t}$$

(35) $$G_{12}\left(t\right)=0.393822+0.043479{\mathrm{e}}^{-t/10000}$$

**Figure 18:**
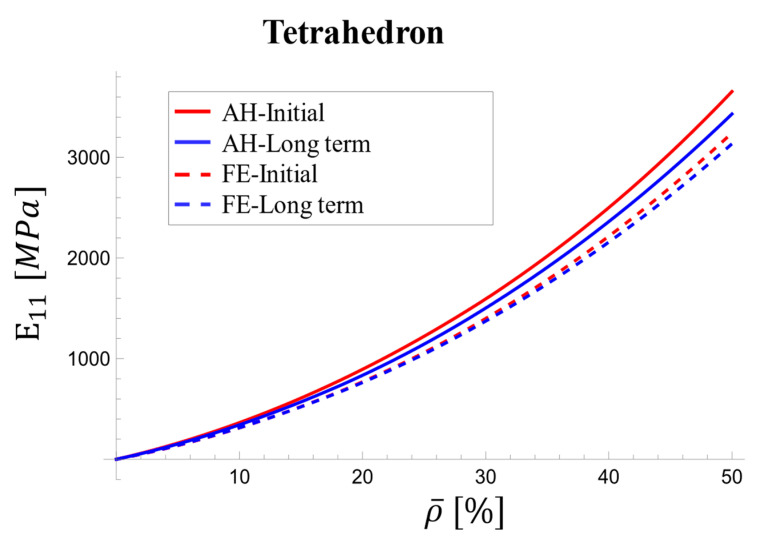
Variations in effective viscoelastic modulus with relative density for tetrahedron-based unit cell at initial stage and over long term.

Figure 18 depicts the variation of the relaxation modulus ($E_{1111}$) with relative density for the tetrahedron-based unit cell. The values were obtained by interpolating from Table 6, which includes data for five densities.

**Figure 19:**
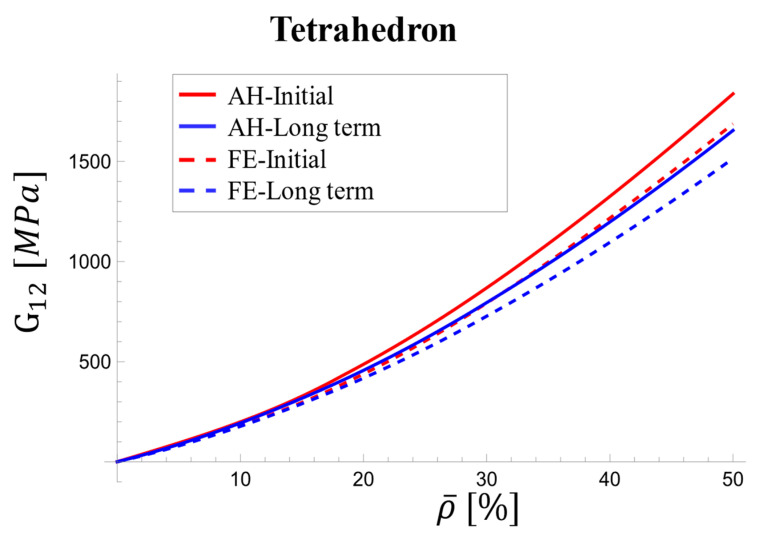
Variations in effective viscoelastic shear modulus with relative density for tetrahedron-based unit cell at initial stage and over long term.

At the loading onset, the AH and FE results exhibit differences ranging from 5.7% and 6.6%. These discrepancies remain relatively constant across densities. In the long term, the difference narrows to 1.5% at 5% density and 3% at 50% density. The discrepancy between the AH and FE results for the viscoelastic shear modulus increased with relative density, particularly at the onset of loading, reaching a maximum of 10% (Figure 19).

**Figure 20:**
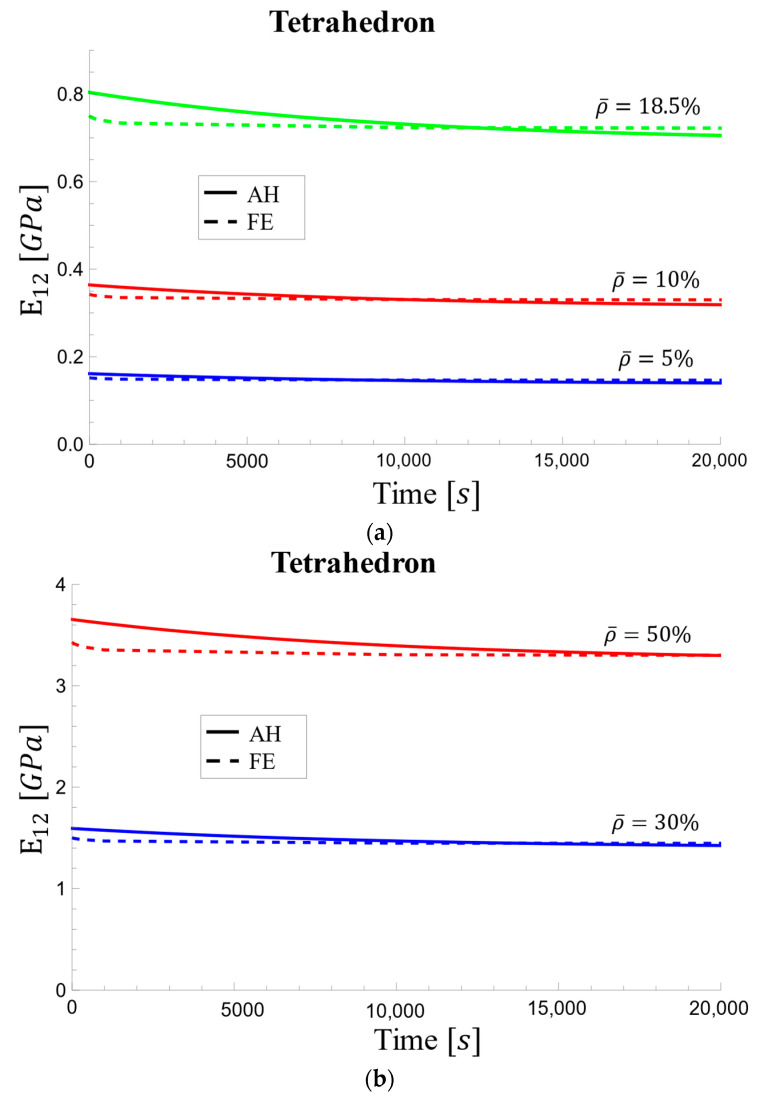
Viscoelastic effective elastic modulus changes over time for tetrahedron-based unit cell for relative densities of (**a**) 5%, 10%, and 18.5% and (**b**) 30% and 50%.

As shown in Figure 20, the rate of decrease is steeper for the AH method, especially at the initial time point. This earlier steady state in the AH method suggests a faster reduction in elastic modulus compared to the FE method, potentially due to the approximations used in the homogenization model. The largest difference is observed at the initial time point for all densities.

**Figure 21:**
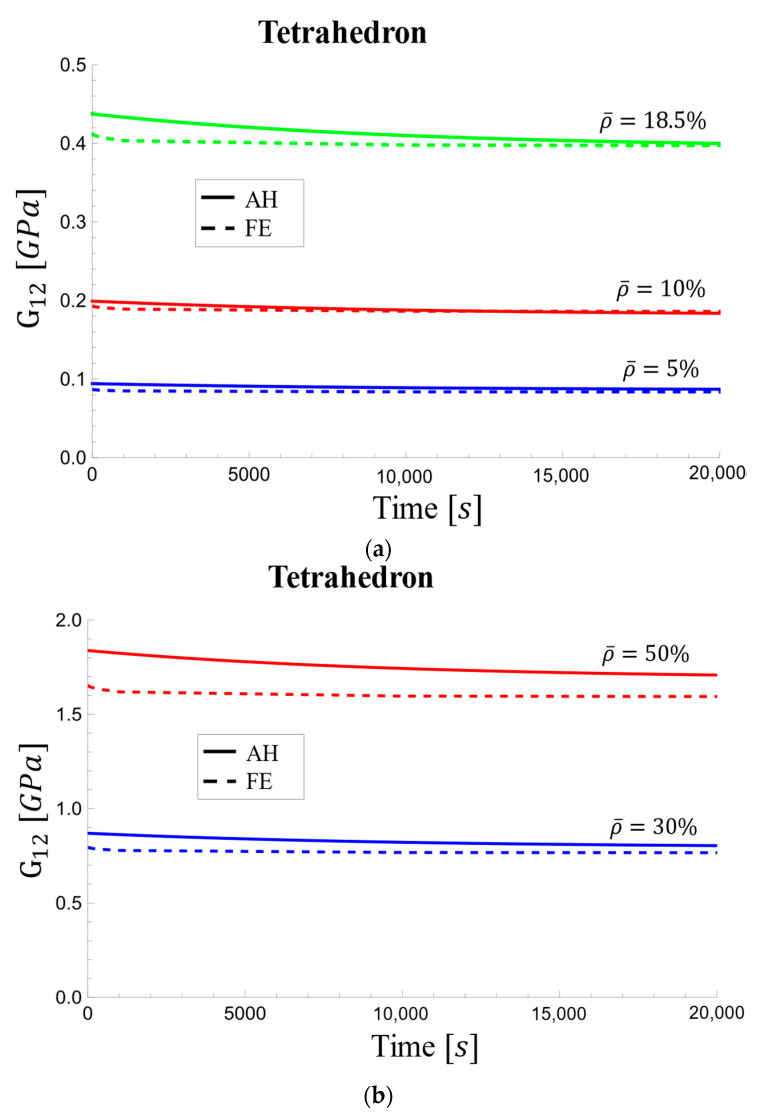
Viscoelastic effective shear modulus changes over time for tetrahedron-based unit cell for relative densities of (**a**) 5%, 10%, and 18.5% and (**b**) 30% and 50%.

Similar to the trends observed for the relaxation modulus (Figure 20), the effective viscoelastic shear modulus also exhibits differences between the AH and FE methods. The AH method yields a steeper decrease compared to the FE method, with the difference becoming less pronounced at higher densities (Figure 21b). Initially, the FE method exhibits a smaller decrease, but a slight decrease continues over time.

#### Poisson’s Ratio

Table 7 presents the effective Poisson’s ratio of the tetrahedron-based unit cell at the initial loading stage and long-term stage.

**Figure 22:**
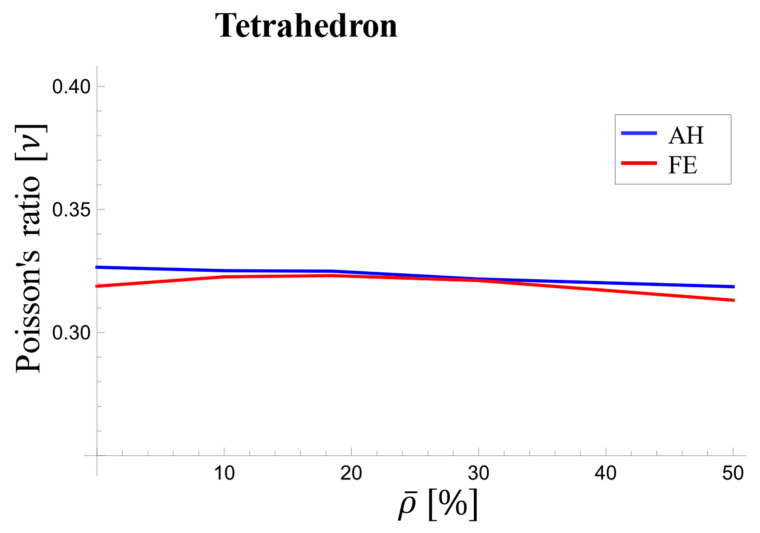
Variations in the effective Poisson’s ratio with relative density at the beginning of loading for the tetrahedron-based unit cell.

Figure 22 depicts the values of Poisson’s ratio for different relative densities at initial loading and early elastic response. The difference between the curves peaked at approximately 1.6% at 5% relative density, approached zero for 30% relative density, and rose to 1.8% for 50% relative density. As observed, Poisson’s ratio exhibits a more or less decreasing trend with increasing density.

**Figure 23:**
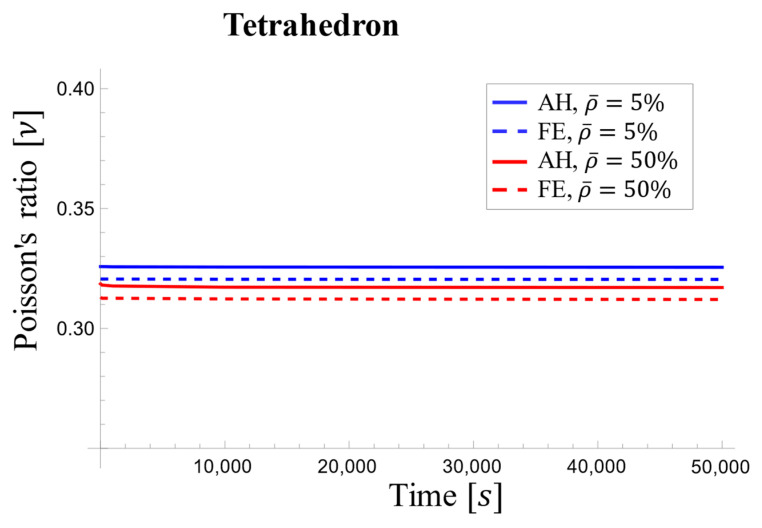
Variations in the effective Poisson’s ratio with time for the tetrahedron-based unit cell.

Figure 23 showcases the negligible variations in Poisson’s ratio over time for both high (50%) and low (5%) relative densities. Therefore, Poisson’s ratio could be considered a constant material property for the tetrahedron-based unit cell in viscoelastic analyses due to its minimal variations over time, as shown in Figure 23.

### 3.3. Performance Comparison: Octet-Truss vs. Tetrahedron-Based Unit Cells

**Figure 24:**
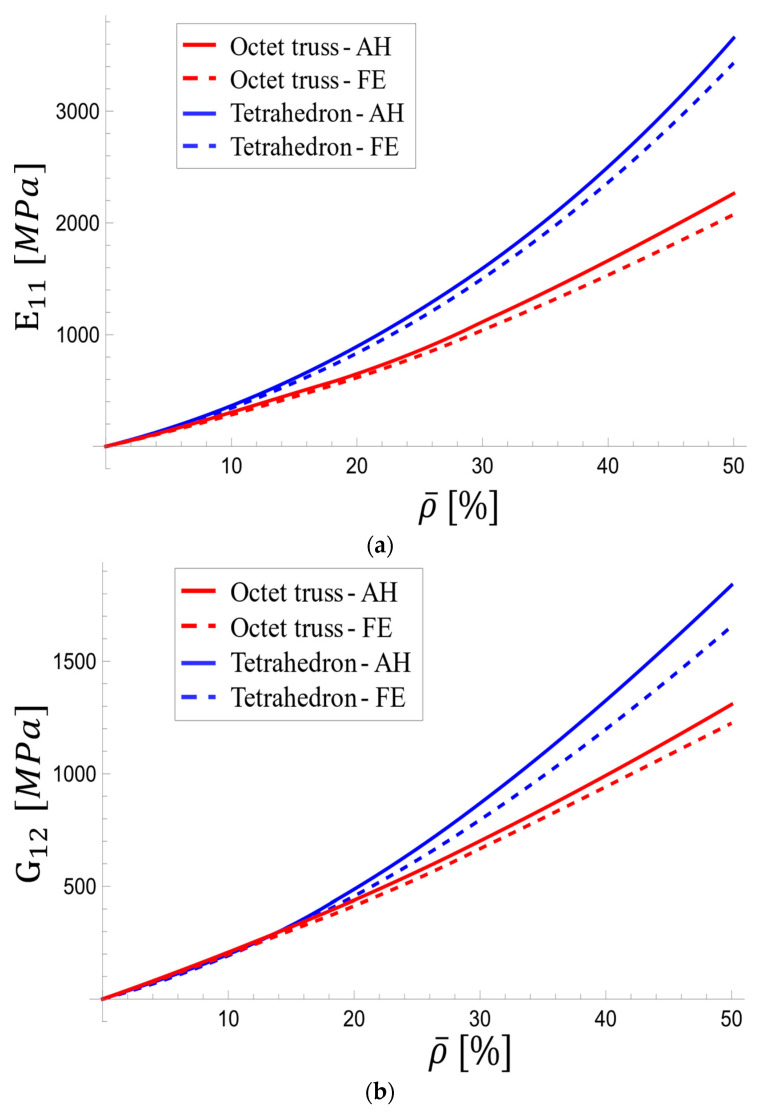
Variations in (**a**) longitudinal and (**b**) shear effective viscoelastic modulus with relative density for the octet-truss and tetrahedron-based unit cells.

The two unit cells, selected for their applicability in medical implants due to their characteristic stretch-dominated deformation behavior, high yield strength, and substantial elastic modulus, exhibit distinct mechanical properties. A comparative analysis of their longitudinal and shear elastic moduli is particularly informative in exploring their performance under various conditions (Figure 24).

Figure 24a reveals that the tetrahedron-based unit cell possessed significantly higher longitudinal modulus than the octet-truss, particularly at high densities. The homogenization method showed that the tetrahedron’s viscoelastic modulus exceeds that of the octet-truss by 13.7% at 5% relative density and 61.5% at 50% relative density. Interestingly, the trend for shear modulus (Figure 24b) was reversed at low densities, with the octet-truss exhibiting a higher value. However, the tetrahedron’s shear modulus increased more rapidly at higher densities, surpassing the octet-truss by 40.5% at 50% relative density. The shear modulus curves intersect at a relative density of ~14%.

**Figure 25:**
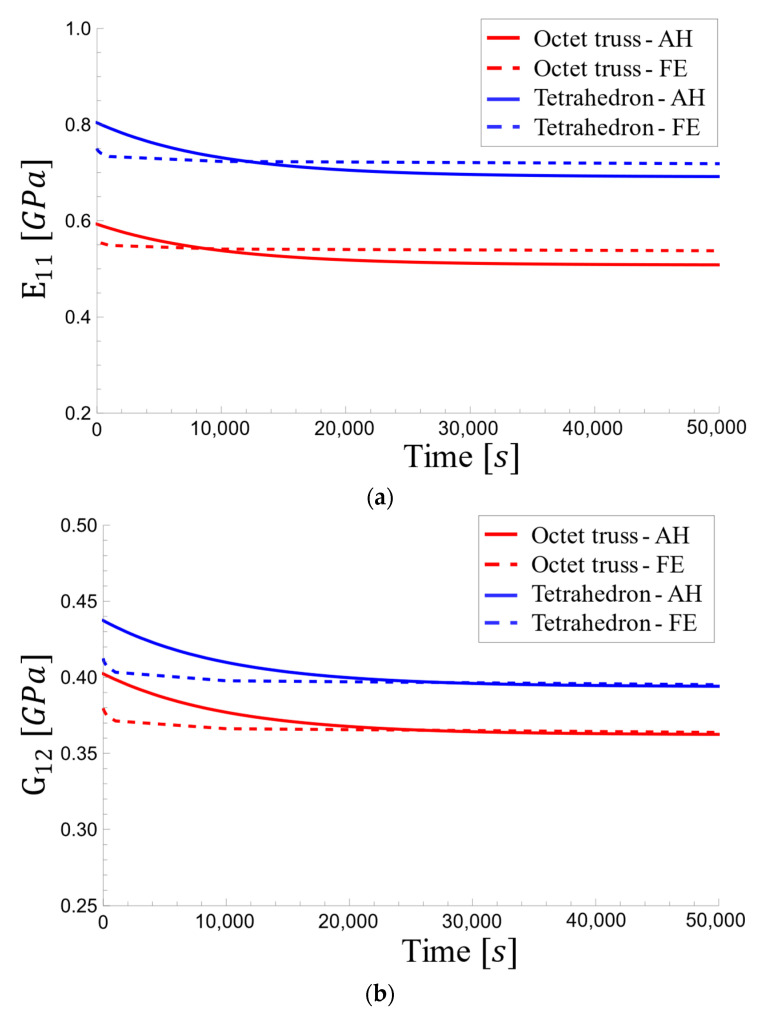
Variations in the effective viscoelastic modulus with time for the octet-truss and tetrahedron-based unit cells at a relative density of 18.5%: (**a**) longitudinal and (**b**) shear moduli.

Figure 25 depicts the transient response of the effective viscoelastic moduli for both octet-truss and tetrahedral-based unit cells at a relative density of 18.5%. They exhibited similar trends of decreasing slope and gradual stabilization over time. As compared to the FE approach, the homogenization method resulted in a steeper slope and earlier steadiness, with the longitudinal and shear moduli approaching constant values after approximately 25,000 s and 35,000 s, respectively.

### 3.4. von Mises Stress Analysis

The von Mises stress analysis provides valuable insights into the stress distribution and relaxation response of two types of unit cells, namely, the octet-truss and tetrahedron-based designs, at two distinct relative densities: 18.5% and 50%. Stress values were evaluated immediately after the application of strain and after a prolonged relaxation period.

#### 3.4.1. Octet-Truss Unit Cell

**Figure 26:**
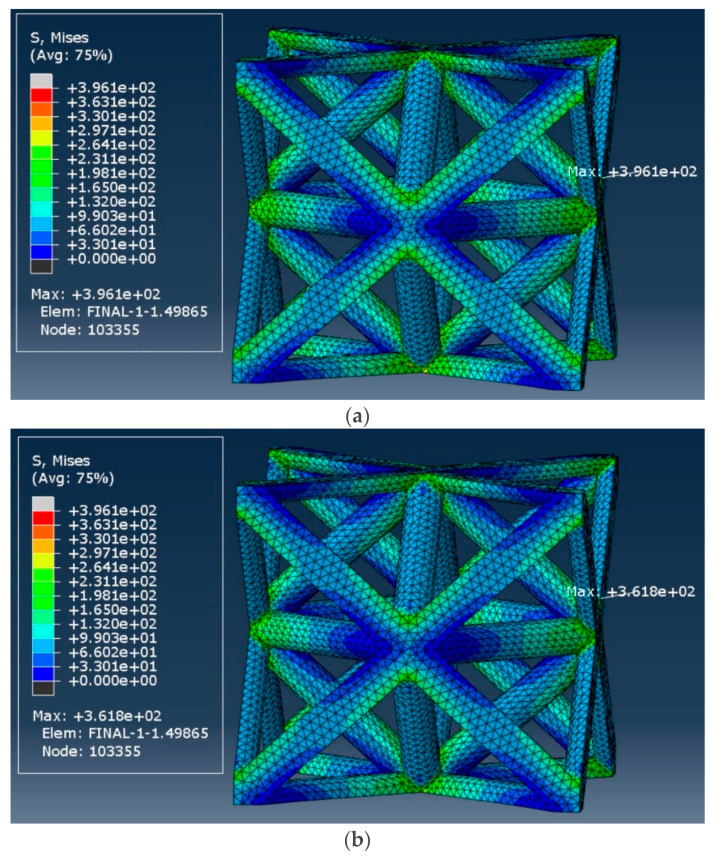
von Mises stress distribution in the octet-truss unit cell with a relative density of 18.5%: (**a**) at the start of strain application, and (**b**) after $10^{7}$ s.

Figure 26 shows the von Mises stress distribution in the octet-truss unit cell with a relative density of 18.5% at two-time instances: (a) immediately after applying the 1% strain and (b) after a long relaxation period ($10^{7}$ s). The maximum stress in the unit cell initially reached 396.1 MPa and inclined to 361.8 MPa in the long term, highlighting the stress relaxation behavior (i.e., 8.7% decrease).

**Figure 27:**
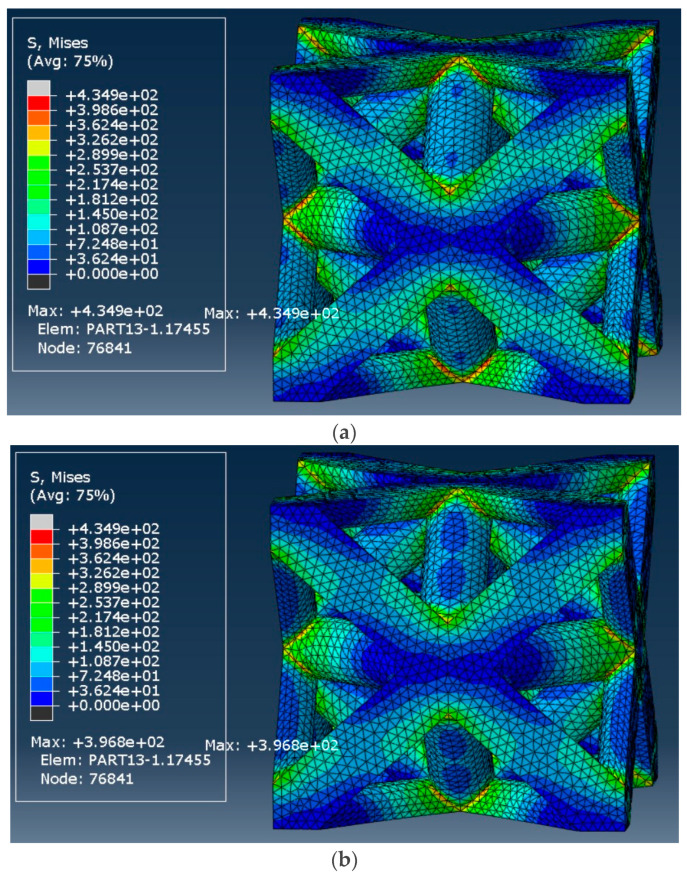
von Mises stress distribution in the octet-truss unit cell with a relative density of 50%: (**a**) at the start of strain application and (**b**) After $10^{7}$ s.

Similarly, Figure 27 represents the von Mises stress distribution for the octet-truss with a 50% relative density. As expected, the material exhibited significant stress relaxation, with the initial stress of 434.9 MPa decreasing to 396.8 MPa in the long term (i.e., 8.8% decrease).

#### 3.4.2. Tetrahedron-Based Unit Cell

**Figure 28:**
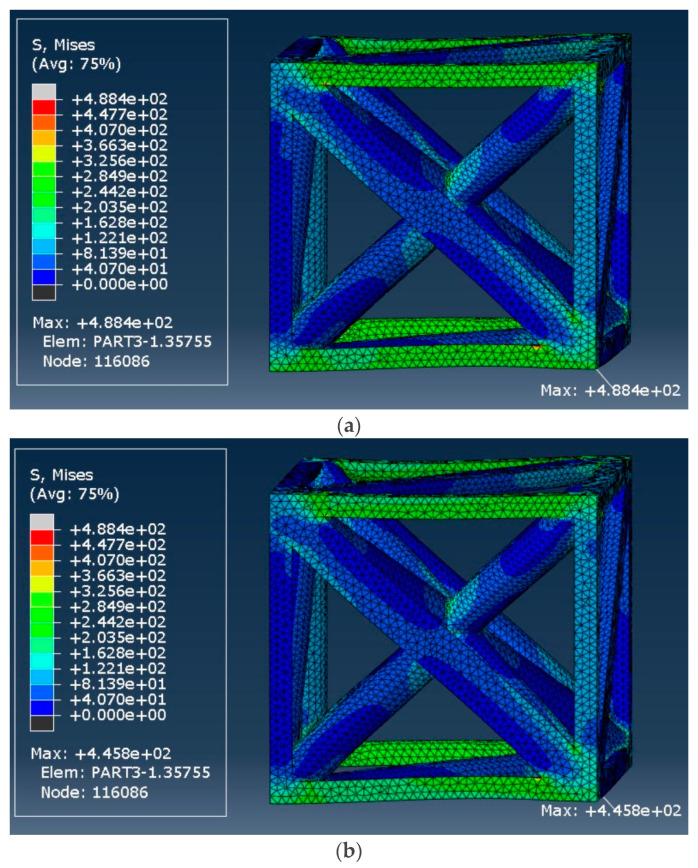
von Mises stress distribution in the tetrahedron-based unit cell with a relative density of 18.5%: (**a**) at the start of strain application and (**b**) After $10^{7}$ s.

The von Mises stress distribution of the tetrahedron-based unit cell with a relative density of 18.5% is shown in Figure 28. The initial stress reached 488.4 MPa, decreasing to 445.8 MPa after a long period (i.e., 8.7% decrease).

**Figure 29:**
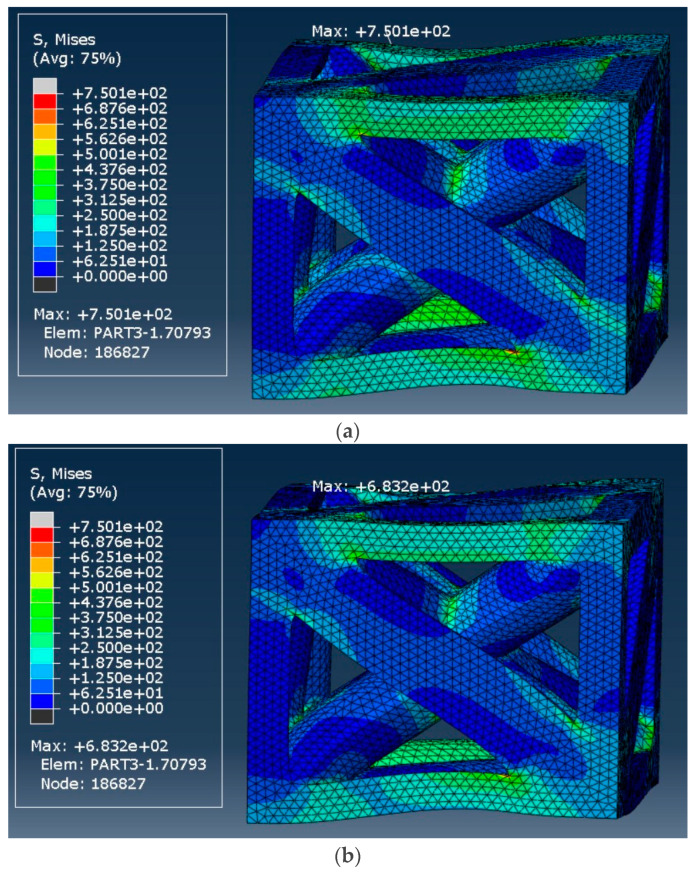
von Mises stress distribution in the tetrahedron-based unit cell with a relative density of 50%: (**a**) at the start of strain application and (**b**) After $10^{7}$ s.

Figure 29 shows the stress distribution of the 50% relative density unit cell. The initial response to longitudinal strain exhibited a maximum stress of 750.1 MPa, which decreased to 683.1 MPa after a long period (i.e., 8.9% decrease). The results show that regardless of the unit cell type and relative density, the decrease in the percentage of stress level remains relatively constant, i.e., ~8.8%.

## 4. Conclusions

This study investigated the effective viscoelastic behavior of two promising unit cells for biomedical fields (tetrahedron-based and octet-truss) for applications demanding both high porosity and strong mechanics. The asymptotic homogenization (AH) method results demonstrated good agreement with those of finite element simulations (ABAQUS), with discrepancies generally less than 10%. The AH results were further validated by comparing them to analytical relationships proposed by other studies and compression experimental tests on octet-truss lattices with different porosity levels.

The main findings of this study are as follows:

- From experimental tests, it was observed that density significantly affects mechanical properties in octet-truss lattices. Both yield strength and elastic modulus increased relatively non-linearly with density. Interestingly, the decrease in elastic modulus (13%) was smaller than the decrease in yield strength (48%) for a relative density reduction from 50% to 30%.
- A strong correlation existed between relative density and the effective elastic modulus of both unit cell designs. The tetrahedron-based structure exhibited significantly higher (longitudinal and shear) modulus compared to the octet-truss, particularly at higher densities. For instance, at 50% relative density, the tetrahedron’s longitudinal and shear moduli surpassed those of the octet-truss by 61.5% and 40.5%, respectively (based on the AH method). This highlights the trade-off between porosity (important for tissue in-growth) and mechanical support. For applications requiring high load-bearing capability, the tetrahedron-based design has shown to be preferable, especially at higher densities.
- Both unit cells exhibited minimal changes in moduli and a near-constant Poisson’s ratio (varying from 0.34 to 0.349 for octet-truss and 0.3258 to 0.3161 for tetrahedron) under the test conditions (PEEK polymer at room temperature). While statistically insignificant, the potential time-dependent stiffness reduction should be considered for long-term simulations.
- Both cell types exhibited stress relaxation over time, indicating their viscoelastic nature. The tetrahedral unit cell exhibited a higher initial stress (750.1 MPa) due to its inherent stiffness compared to the octet-truss (488.4 MPa).

## Figures and Tables

**Table 1:** Somerville’s irregular tetrahedron number 3 properties [54].

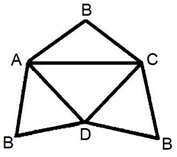		
Side	Relative Length	Angle
AB	$\sqrt{3}$	60°
AC	$2\sqrt{2}$	90°
AD	2	45°
BC	$\sqrt{3}$	60°
BD	$\sqrt{3}$	120°
CD	2	45°

**Table 2:** Relaxation longitudinal and shear modulus values in different time intervals.

Time [s]	E [GPa]	$G\;\left[GPa\right]$	Time [s]	$E\;\left[GPa\right]$	$G\;\left[GPa\right]$
0	21.65	7.637	2000	21.09	7.466
10	21.56	7.625	$10^{4}$	20.87	7.395
20	21.49	7.602	$10^{5}$	20.57	7.299
100	21.37	7.557	$10^{6}$	20.33	7.214
200	21.31	7.539	$10^{7}$	20.11	7.137
1000	21.17	7.491	Long-term	19.80	7.020

**Table 3:** Viscoelastic modulus components.

Indices	Boundary Condition	Effective Modulus Components
k= l=2	$\varepsilon_{22}^{0}=1$	${\bar{E}}_{1122}^{h},\;{\bar{E}}_{2222}^{h},\;{\bar{E}}_{3322}^{h}$
k= l=3	$\varepsilon_{33}^{0}=1$	${\bar{E}}_{1133}^{h},\;{\bar{E}}_{2233}^{h},\;{\bar{E}}_{3333}^{h}$
k=1, l=2	$2\varepsilon_{12}^{0}=1$	${\bar{E}}_{1212}^{h}$
k=1, l=3	$2\varepsilon_{13}^{0}=1$	${\bar{E}}_{1313}^{h}$
k=2, l=3	$2\varepsilon_{23}^{0}=1$	${\bar{E}}_{2323}^{h}$

**Table 4:** The effective relaxation moduli calculated at the beginning of loading and after a long period for the octet-truss unit cell.

	$\bar{\rho }\;(\%)$	$E_{11}\;[MPa]$			$G_{12}\;[MPa]$		
		AH	FE	Deshpande et al. [67]	AH	FE	Deshpande et al. [67]
Beginning of loading	5	141.8	133.5	180.4	101.4	96.3	90.2
	10	305	283	360.8	208.5	199.1	180.4
	18.5	593	561.4	667.54	402.3	379.7	333.8
	30	1115.8	1040.6	1082.5	701.3	667.2	541.2
	50	2263.1	2075.2	1804.2	1308.1	1224.6	902.1
After a long period	5	120.6	122.1	165	92	88.1	82.5
	10	260.8	258.9	330	190.2	182.1	165
	18.5	507.9	513.4	610.5	362.2	347.3	305.25
	30	961.8	951.7	990	646.7	610.3	495
	50	1971.7	1897.5	1650	1154.7	1120.3	825

**Table 5:** Effective Poisson’s ratio at the beginning of loading and after a long period for the octet-truss unit cell.

$\bar{\rho }\;(\%)$	Beginning of Loading		After a Long Period	
	AH	FE	AH	FE
5	0.3489	0.3392	0.3485	0.3389
10	0.3472	0.3401	0.3467	0.3397
18.5	0.3476	0.3391	0.3469	0.3384
30	0.3415	0.3362	0.3409	0.3353
50	0.3408	0.3303	0.3401	0.3288

**Table 6:** The effective relaxation moduli calculated at the beginning of loading and after a long period for the tetrahedron-based unit cell.

	$\bar{\rho }\;(\%)$	$E_{11}\;[MPa]$			$G_{12}\;[MPa]$		
		AH	FE	Deshpande et al. [67]	AH	FE	Deshpande et al. [67]
Beginning of loading	5	94.1	86.7	161.3	152.1	94.1	86.7
	10	199.1	193.2	364	342.9	199.1	193.2
	18.5	437.3	412.5	803.9	750.6	437.3	412.5
	30	868.7	795.1	1593.2	1502.7	868.7	795.1
	50	1838	1655.1	3654.5	3431.2	1838	1655.1
After a long period	5	85.8	79.3	137	139.1	85.8	79.3
	10	181.1	176.7	312.3	313.5	181.1	176.7
	18.5	393.8	377.3	691.3	686.2	393.8	377.3
	30	792.8	727.4	1398.9	1373.5	792.8	727.4
	50	1687.6	1515.2	3243	3134.9	1687.6	1515.2

**Table 7:** Effective Poisson’s ratio at the beginning of loading and after a long period for the tetrahedron-based unit cell.

$\bar{\rho }\;(\%)$	Beginning of Loading		After a Long Period	
	AH	FE	AH	FE
5	0.3258	0.3207	0.3254	0.3204
10	0.3251	0.3226	0.3247	0.3222
18.5	0.3249	0.3231	0.3246	0.3225
30	0.3217	0.3211	0.3208	0.32
50	0.3186	0.3131	0.3161	0.3113

## Data Availability

The original contributions presented in this study are included in the article/supplementary material. Further inquiries can be directed to the corresponding author.

## Supplementary Materials

The following supporting information can be downloaded at: https://www.mdpi.com/article/10.3390/ma17235865/s1, Figure S1: Stress-strain curve results from (a) tensile and (b) compressive tests on PLA; Figure S2: Effective Modulus vs. Number of Elements (Octet Truss, Homogenization); Figure S3: Effective Modulus vs. Number of Elements (Tetrahedral, Homogenization); Table S1: Tensile test results; Table S2: Compression test results; Table S3: Viscoelastic modulus ${(E}_{11})$ for Various Mesh Refinements (Octet Truss Unit Cell, 18.5% Relative Density, Homogenization Method); Table S4: Viscoelastic modulus ${(E}_{11})$ for Various Mesh Refinements (Octet Truss Unit Cell, 18.5% Relative Density, Finite Element Method using Abaqus); Table S5: Viscoelastic modulus ${(E}_{11})$ for Various Mesh Refinements (Tetrahedron-Based Unit Cell, 18.5% Relative Density, Homogenization Method); Table S6: Viscoelastic modulus ${(E}_{11})$ for Various Mesh Refinements (Tetrahedron-Based Unit Cell, 18.5% Relative Density, Finite Element Method using Abaqus).
