# Supplementary material for: Viscoelastic Behavior of Cellular Biomaterials Based on Octet-Truss and Tetrahedron Topologies
Source: Materials (Basel). 2024 Nov 29;17(23):5865. doi: 10.3390/ma17235865 (PMC11643608; doi:10.3390/ma17235865)
Supplement: Supplementary file 1 [file materials-17-05865-s001.zip › materials-3323642-supplementary.pdf]

Supplementary Materials

S.1. Tensile and Compression tests

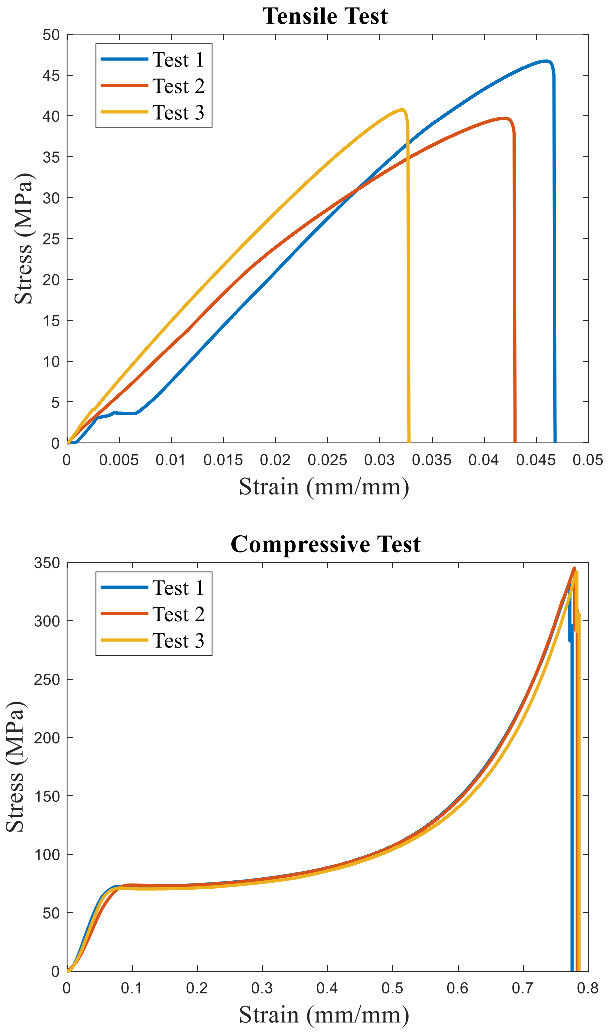

Figure S1: The stress-strain curve results of (a) tensile and (b) compressive tests on PLA

Table S1: Tensile test results

	E	Yield Strength	Failure Stress	Failure Strain	Tangent Modulus
Test 1	1.3 GPa	42 MPa	46 MPa	0.046	0.75 GPa
Test 2	1.2 GPa	32 MPa	40 MPa	0.043	0.61 GPa
Test 3	1.4 GPa	36 MPa	41 MPa	0.032	1.01 GPa
Mean	1.3 ±0.113 GPa	37 ±5.696 GPa	42 ±3.638 GPa	0.040 ±0.00834	0.79 ±0.23 GPa

Table S2: Compression test results

	E	Yield Strength
Test 1	1.6 GPa	60 MPa
Test 2	1.1 GPa	55 MPa
Test 3	1.5 GPa	63 MPa
Mean	1.4 ±0.299 GPa	59 ±4.573 GPa

S.2. Mesh sensitivity analysis

S.2.1. Octet truss unit cell

This section investigates the influence of mesh refinement on the accuracy of the homogenization method for an octet truss unit cell with a relative density of 18.5%. Four progressively finer meshes were generated using first-order tetrahedral elements (C3D4). The resulting viscoelastic moduli are summarized in Table S3.

Table S3: Viscoelastic modulus (E_{11}) for Various Mesh Refinements (Octet Truss Unit Cell, 18.5% Relative Density, Homogenization Method)

Number of elements (C3D4)	E_{11} [MPa]	
	Beginning of loading	After a long period
1263	596.3	510.3
2766	594.1	508.7
4812	593.0	507.9
7059	592.6	507.6

Due to computational resource limitations, first-order elements (C3D4) were initially used. Table S3 demonstrates that 4812 elements provided sufficient accuracy, as shown in Figure S2 (based on the initial loading modulus). Further refinement yielded negligible changes in results.

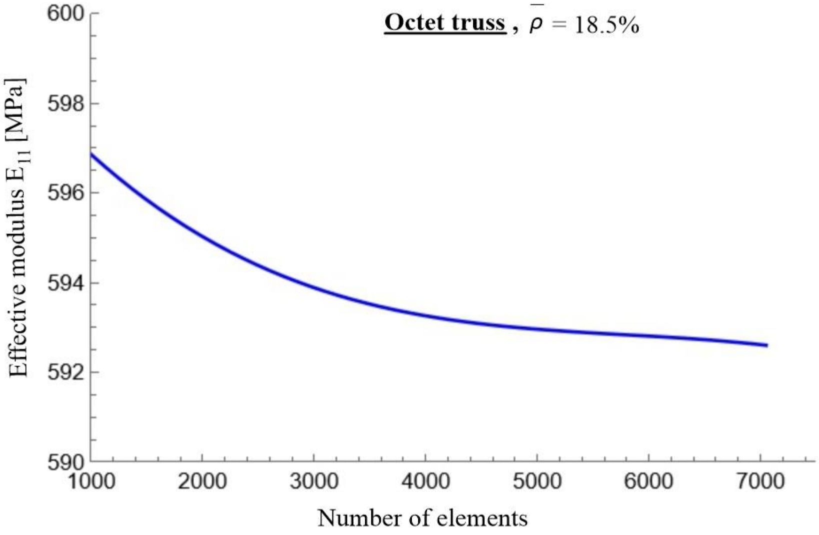

Figure S2: Effective Modulus vs. Number of Elements (Octet Truss, Homogenization)

Abaqus software allows for faster solution speeds, enabling the use of finer meshes with primarily second-order tetrahedral elements (C3D10) for improved accuracy. C3D4 elements were only used in one instance for comparison. Table S4 presents the effective viscoelastic moduli obtained using four mesh densities of C3D10 elements. The results indicate that a mesh exceeding 50,000 elements is necessary for acceptable accuracy.

Table S4: Viscoelastic modulus (E_{11}) for Various Mesh Refinements (Octet Truss Unit Cell, 18.5% Relative Density, Finite Element Method using Abaqus)

Elements	E_{11} [MPa]	
	Beginning of loading	After a long period
14573 of C3D10	564.13	515.91
52553 of C3D10	561.36	513.41
106400 of C3D10	560.28	512.53
71931 of C3D4	569.44	519.86

S.2.2. Tetrahedron-based unit cell

Following the same approach, the effective axial viscoelastic modulus for a tetrahedron-based unit cell with 18.5% relative density was calculated using the homogenization method with C3D4 elements. The results for four different meshing resolutions are presented in Table S5.

Table S5: Viscoelastic modulus (E_{11}) for Various Mesh Refinements (Tetrahedron-Based Unit Cell, 18.5% Relative Density, Homogenization Method)

Number of elements (C3D4)	E_{11} [MPa]	
	Beginning of loading	After a long period
1043	806.7	693.2
2367	804.5	691.7
4129	803.9	691.3
6395	803.6	691.1

As shown in Table S5 and Figure S3 (effective modulus vs. number of elements), a mesh with 4129 elements provides good accuracy, with further refinement having a negligible effect on the results.

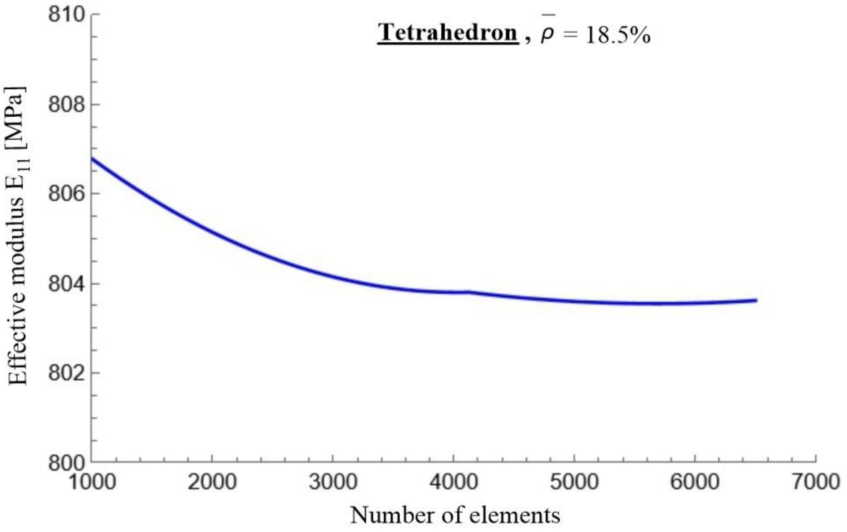

Figure S3: Effective Modulus vs. Number of Elements (Tetrahedral, Homogenization)

For the finite element analysis in Abaqus, second-order tetrahedral elements (C3D10) were employed. Table S6 includes the results for four mesh densities, along with one case using C3D4 elements for comparison.

Table S6: Viscoelastic modulus (E_{11}) for Various Mesh Refinements (Tetrahedron-Based Unit Cell, 18.5% Relative Density, Finite Element Method using Abaqus)

Elements	E_{11} [MPa]	
	Beginning of loading	After a long period
16467 of C3D10	753.1	688.7
51260 of C3D10	750.9	686.6
94022 of C3D10	750.6	686.2
68351 of C3D4	757.3	691.4
